# Comparative ultrastructure of cells and cuticle in the anterior chamber and papillate region of *Porcellioscaber* (Crustacea, Isopoda) hindgut

**DOI:** 10.3897/zookeys.801.22395

**Published:** 2018-12-03

**Authors:** Urban Bogataj, Monika Praznik, Polona Mrak, Jasna Štrus, Magda Tušek-Žnidarič, Nada Žnidaršič

**Affiliations:** 1 Department of Biology, Biotechnical Faculty, University of Ljubljana, Večna pot 111, 1000 Ljubljana, Slovenia; 2 Faculty of Chemistry and Chemical Technology, University of Ljubljana, Večna pot 113, 1000 Ljubljana, Slovenia; 3 National Institute of Biology, Večna pot 111, 1000 Ljubljana

**Keywords:** cell junctions, digestive system, extracellular matrix, ion transporting epithelium, plasma membrane labyrinth

## Abstract

Isopod hindgut consists of two anatomical and functional parts, the anterior chamber, and the papillate region. This study provides a detailed ultrastructural comparison of epithelial cells in the anterior chamber and the papillate region with focus on cuticle ultrastructure, apical and basal plasma membrane labyrinths, and cell junctions. Na^+^/K^+^-ATPase activity in the hindgut epithelial cells was demonstrated by cytochemical localisation. The main difference in cuticle ultrastructure is in the thickness of epicuticle which is almost as thick as the procuticle in the papillate region and only about one sixth of the thickness of procuticle in the anterior chamber. The apical plasma membrane in both hindgut regions forms an apical plasma membrane labyrinth of cytoplasmic strands and extracellular spaces. In the papillate region the membranous infoldings are deeper and the extracellular spaces are wider. The basal plasma membrane is extensively infolded and associated with numerous mitochondria in the papillate region, while it forms relatively scarce basal infoldings in the anterior chamber. The junctional complex in both hindgut regions consists of adherens and septate junctions. Septate junctions are more extensive in the papillate region. Na^+^/K^+^-ATPase was located mostly in the apical plasma membranes in both hindgut regions. The ultrastructural features of hindgut cuticle are discussed in comparison to exoskeletal cuticle and to cuticles of other arthropod transporting epithelia from the perspective of their mechanical properties and permeability. The morphology of apical and basal plasma membranes and localisation of Na^+^/K^+^-ATPase are compared with other arthropod-transporting epithelia according to different functions of the anterior chamber and the papillate region.

## Introduction

The digestive system in terrestrial isopods is composed of a foregut, hindgut, and hepatopancreas (midgut glands). The foregut and hindgut are of ectodermal origin and form the entire alimentary canal, thus the blind-ending tubules of hepatopancreas connected to the foregut represent the only endodermal part of digestive system (Vernon et al. 1974, Hassall and Jennings 1975, Bettica et al. 1987, Storch and Štrus 1989, Štrus et al. 2008). In some amphibious species, a short segment of endodermal midgut is located between the foregut and the hindgut (Štrus et al. 1995). The hindgut is divided into an anterior chamber, papillate region and short rectum separated from the papillate region by a muscular sphincter. In the anterior chamber of terrestrial isopods, the folded dorsal hindgut wall forms a dorsal longitudinal fold termed typhlosole, together with two typhlosole channels. In the papillate region the dome-shaped basal parts of epithelial cells bulge into haemocoel between the longitudinal and circular muscles surrounding the hindgut (Hassal and Jennings 1975, Hames and Hopkin 1989, Štrus et al. 1995). Hames and Hopkin (1989) reported that the anterior chamber is the site where digestion of food mixed with digestive enzymes takes place, while the papillate region is involved in compaction of faecal pellets and removal of water.

The hindgut epithelium is mono-layered and lined by a chitinous cuticle on the luminal side. Basal parts of the epithelial cells are supported by a basal lamina and are exposed to haemolymph. The hindgut cuticle is thin and consists of two distinct layers, the electron dense epicuticle facing the lumen and the electron lucent procuticle beneath. Posteriorly directed cuticular spines are present at the surface of epicuticle (Vernon et al. 1974, Palackal et al. 1984, Storch and Štrus 1989, Mrak et al. 2015). The main ultrastructural characteristics of hindgut epithelial cells are the extensively infolded apical and basal plasma membranes, numerous mitochondria associated with membrane infoldings, abundant apico-basally oriented microtubules, and extensive septate junctions (Vernon et al. 1974, Coruzzi et al. 1982, Palackal et al. 1984, Storch and Štrus 1989). Previous ultrastructural studies have provided knowledge concerning the general ultrastructure of hindgut epithelial cells in isopods. The specific gaps to be addressed are the differences in the ultrastructure of hindgut epithelial cells in the two main hindgut regions, the anterior chamber, and the papillate region.

Notwithstanding the presence of apical chitinous cuticle, the ultrastructural characteristics indicate that the hindgut epithelium is involved in various transport processes. The epithelium of the anterior chamber was reported to function in the absorption of food material in addition to the hepatopancreas, which represents the main site of food absorption (Hryniewiecka-Szyfter and Storch 1986, Hames and Hopkin 1989). Extensively infolded apical and basal plasma membranes associated with numerous mitochondria, abundant microtubular network and extensive septate junctions indicate the involvement of papillate region epithelium in ion and water transport and thus in osmoregulation (Vernon et al. 1974, Coruzzi et al. 1982, Palackal et al. 1984). Ultracytochemical localisation of Na^+^/K^+^-ATPase activity in the hindgut of *Armadilloofficinalis* has shown Na^+^/K^+^-ATPase activity in the apical or basolateral plasma membrane infoldings (Warburg and Rosenberg 1989).

The aim of the present study is ultrastructural characterisation, quantification of the selected characters and comparison of the hindgut epithelial cells in the anterior chamber and the papillate region of terrestrial isopod *Porcellioscaber* to upgrade previous knowledge and to get additional insight into the hindgut functional morphology. The selected ultrastructural features for a detailed analysis were: (i) cuticle, (ii) apical and basal plasma membrane labyrinths, and (iii) cell junctions. A comparative investigation of the selected ultrastructural features in the anterior chamber and papillate region epithelium is presented, including quantitative evaluation of the selected morphological characteristics, and the possible functional implications are discussed. A method for cytochemical localisation of Na^+^/K^+^-ATPase activity in the hindgut of strictly terrestrial isopod *P.scaber* was used to demonstrate ion fluxes in the hindgut epithelium of intermoult and postmoult specimens.

## Materials and methods

### Sample preparation and imaging

A laboratory culture of *P.scaber* was maintained in a glass terrarium with a ground cover of soil and leaf litter. Animals were bred at 25 °C, in high humidity and a 12h light/12h dark cycle. In this study the hindgut samples of seven adult intermoult animals were analysed at the levels of light and electron microscopy. Three animals were anesthetised by cooling and dissected in a physiological solution (0.9% NaCl). The hindguts were isolated and fixed in 2.5 % glutaraldehyde in 0.1 M HEPES buffer (pH 7.2). Four animals were anesthetised with diethyl ether, dissected in a solution of 2 % paraformaldehyde and 2.5% glutaraldehyde in 0.1 M HEPES buffer (pH 7.2) and isolated hindguts were fixed in the same fixative solution. After fixation, all samples were rinsed with 0.1 M HEPES buffer and post-fixed in 1 % OsO_4_. Subsequently the samples were rinsed again with 0.1 M HEPES buffer. Before the embedding samples were dehydrated in a graded series of ethanol (50 %, 70 %, 80 %, 90 %, and 100 % ethanol) and transferred to absolute acetone. After the dehydration, samples were infiltrated and embedded in Agar 100 resin. Resin-embedded samples were polymerised in embedding moulds for 48 h at 60 °C. Semithin and ultrathin sections were cut with glass and diamond knives on Reichert Ultracut S ultramicrotome (Leica). Semithin sections were stained with Azure II – Methylene Blue and imaged with AxioImager Z.1 light microscope (Zeiss). Microscopic images were acquired with a HRc Axiocam camera using Axiovision software. Ultrathin sections were contrasted with 4 % uranyl acetate and 10 % lead citrate and examined with a Philips CM100 transmission electron microscope. Microscopic images were acquired with Bioscan 792 and Orius 200 (Gatan) cameras using Digital Micrograph software.

### Measurements of selected morphological characteristics

**Figure 1.**
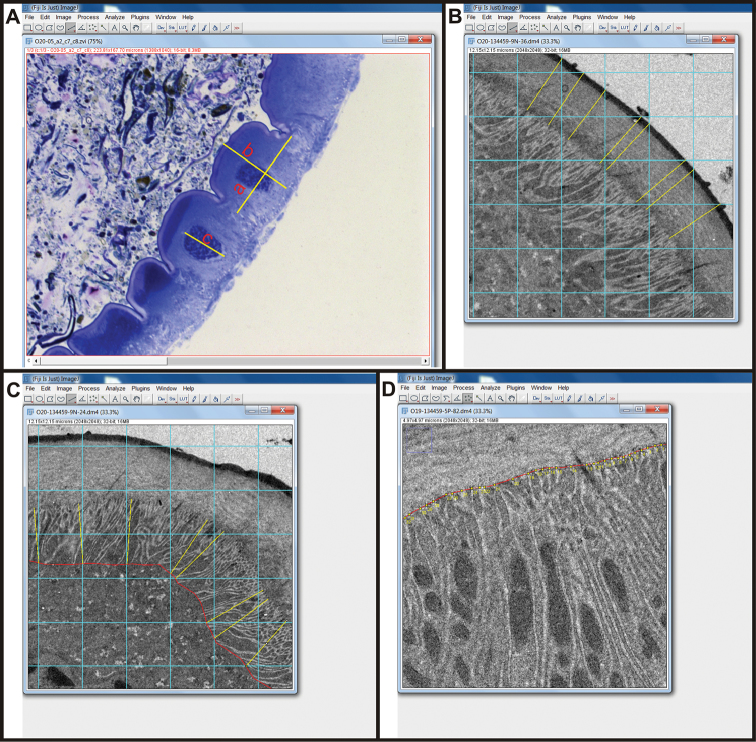
Measurements in ImageJ/Fiji. **A** Measurements of cell width (line a), height (line b) and nucleus diameter (line c) **B** Measurements of cuticle and basal lamina thickness. Cuticle and basal lamina thickness (yellow lines) were measured at intersections of grid lines (blue grid) with cuticle/basal lamina surface **C** Measurements of membrane labyrinths depth. Membrane labyrinth depths (yellow lines) were measured at intersections of grid lines (blue grid) with the outline of membrane labyrinth edge (red line) **D** Measurements of spatial density of membrane infoldings. The length of apical/basal surface outline (red line) was measured and the infoldings along the outline (yellow points) were counted.

Measurements of cell size and cell nuclei diameter, cuticle and basal lamina thicknesses, depth of membrane labyrinths and spatial density of membrane infoldings were carried out with Fiji/ImageJ software for processing and analysis of digital micrographs. Cell height and width and largest cell nucleus diameter were measured on light micrographs with a straight-line tool in Fiji (Fig. 1A). Measurements were done on seven individual animals. In each animal we measured 7–29 cells from the anterior chamber and from the papillate region. Only cells sectioned across their cell nucleus were measured. In the Results section, measurements are presented graphically with a scatter plot depicting the median values of cell width, cell height and cell nucleus diameter in the anterior chamber against median values in the papillate region of seven animals. In the supplementary information (SI), individual measurements from the two hindgut regions of individual animals are presented graphically with boxplots accompanied by stripcharts. Measurements of cuticle and basal lamina thicknesses and membrane labyrinths depths were done on 3 individual animals. For each animal 7–71 measurements were made on 1–4 cells from the anterior chamber and from the papillate region. For measurements of cuticle and basal lamina thicknesses overlays of multipurpose grid on the electron micrographs were made in Fiji. The multipurpose grid is available as macro at: https://imagej.nih.gov/ij/macros/Multipurpose_grid.txt. The thicknesses of the cuticle and of the basal lamina were measured at the intersections of grid lines with the cuticle/basal lamina surface. Measurements were made perpendicular to the cuticle/basal lamina surface with a straight-line tool (Fig. 1B). In the Results section, measurements are presented graphically with stripcharts depicting median values of cuticle thickness and basal lamina thickness in cells from the anterior chamber and the papillate region of 3 animals. In the SI, individual measurements on cells from the two hindgut regions of each animal are presented graphically with boxplots accompanied by stripcharts. For measurements of apical and basal membrane labyrinth depths the edges of the membrane labyrinths were outlined on electron micrographs with segmented line tool in Fiji and overlays of multipurpose grid on micrographs were made. Membrane labyrinth depth was measured at intersections of grid lines with the outline of membrane labyrinth edge. Measurements were made perpendicular to the apical/basal cell surface with a straight-line tool (Fig. 1C). In the Results section, measurements are presented graphically with scatter plots depicting the median values of apical labyrinth depth against median values of basal labyrinth depth in cells from the anterior chamber and the papillate region of three animals. In the SI, individual measurements on cells from the two hindgut regions of each animal are presented graphically with boxplots accompanied by stripcharts. Measurements of spatial density of membrane infoldings were done on three individual animals. For each animal 1–6 measurements were made on 1–4 cells from the anterior chamber and from the papillate region. In Fiji the apical/basal cell surface were outlined with a segmented line tool. The length of outline was measured and the number of membrane infoldings along the outline was counted (Fig. 1D). The density of membrane infoldings per 1 µm of apical/basal cell surface was calculated. In the Results section, measurements are presented graphically with scatter plots depicting median values of apical infolding density against median values of basal infolding density in cells from the anterior chamber and the papillate region of three animals. In the SI, individual measurements on cells from the two hindgut regions of each animal are presented graphically with stripcharts. All plots were done in RStudio.

### Na+/K+-ATPase activity

Ultracytochemical localisation of ouabain-sensitive Na^+^/K^+^-ATPase activity (K^+^-NPPase) in the hindgut of *P.scaber* was performed in postmoult and intermoult adults, using the method of Mayahara et al. (1980). Adult postmoult and intermoult specimens of *P.scaber* were dissected and the hindguts were isolated and cut into small pieces of the anterior and papillate parts. Tissue samples were fixed in 0.5 % glutaraldehyde and 2 % paraformaldehyde in 0.1 M sodium cacodylate buffer (pH 7.4). After fixation tissue samples were incubated for 20 min at 25 °C in a medium containing 250 mM glycine/KOH buffer (pH 9), 4 mM lead citrate, 25 % DMSO, 10 mM p-nitrophenylphosphate (p-NPP), and 2.5 mM levamisole. Tissue samples for controls were incubated in 1) a substrate-free (p-NPP) medium, 2) a medium in which K^+^ ions were replaced by Na^+^ ions and 3) a medium containing 10 mM ouabain, an inhibitor of K^+^-NPPase activity. Tissue samples were then postfixed in 1 % osmium tetroxide and embedded in Spurr resin. Unstained ultrathin sections were examined with a Gatan Bioscan 972 camera attached to a TEM Philips CM 100.

## Results

### Histological structure

**Figure 2.**
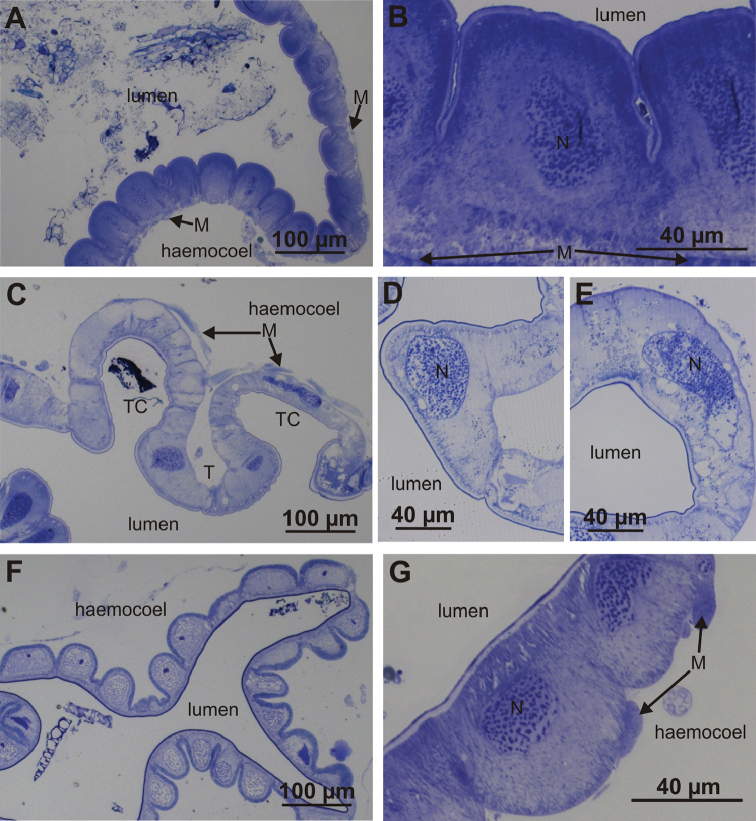
Histological structure of the hindgut epithelium in the anterior chamber and papillate region. **A** Ventral-lateral epithelium in the anterior chamber **B** Apical parts of ventral and lateral epithelial cells are bulging into the hindgut lumen **C** Dorsal epithelium in the anterior chamber forms typhlosole (T) and two typhlosole channels (TC) **D** Epithelial cell of typhlosole **E** Epithelial cell of typhlosole channel **F** Hindgut epithelium in the papillate region **G** Basal parts of epithelial cells in the papillate region are bulging into haemocoel. Abbreviations: M – muscles, N – cell nucleus.

Cells forming the ventral and lateral hindgut wall of anterior chamber are isodiametric and dome-shaped, their apical parts protruding into the lumen of the anterior chamber (Fig. 2A, B). The dorsal epithelial cells that build the typhlosole and typhlosole channels are flatter and wider than cells of the ventral and lateral walls (Fig. 2C, D, E). In the papillate region, epithelial cells are isodiametric, and in contrast to those in the anterior chamber, display dome-shaped basal parts, which protrude into the haemocoel (Fig. 2F, G). Muscle layers are present on the basal side of epithelium in both hindgut regions and are more abundant in the anterior chamber.

**Figure 3.**
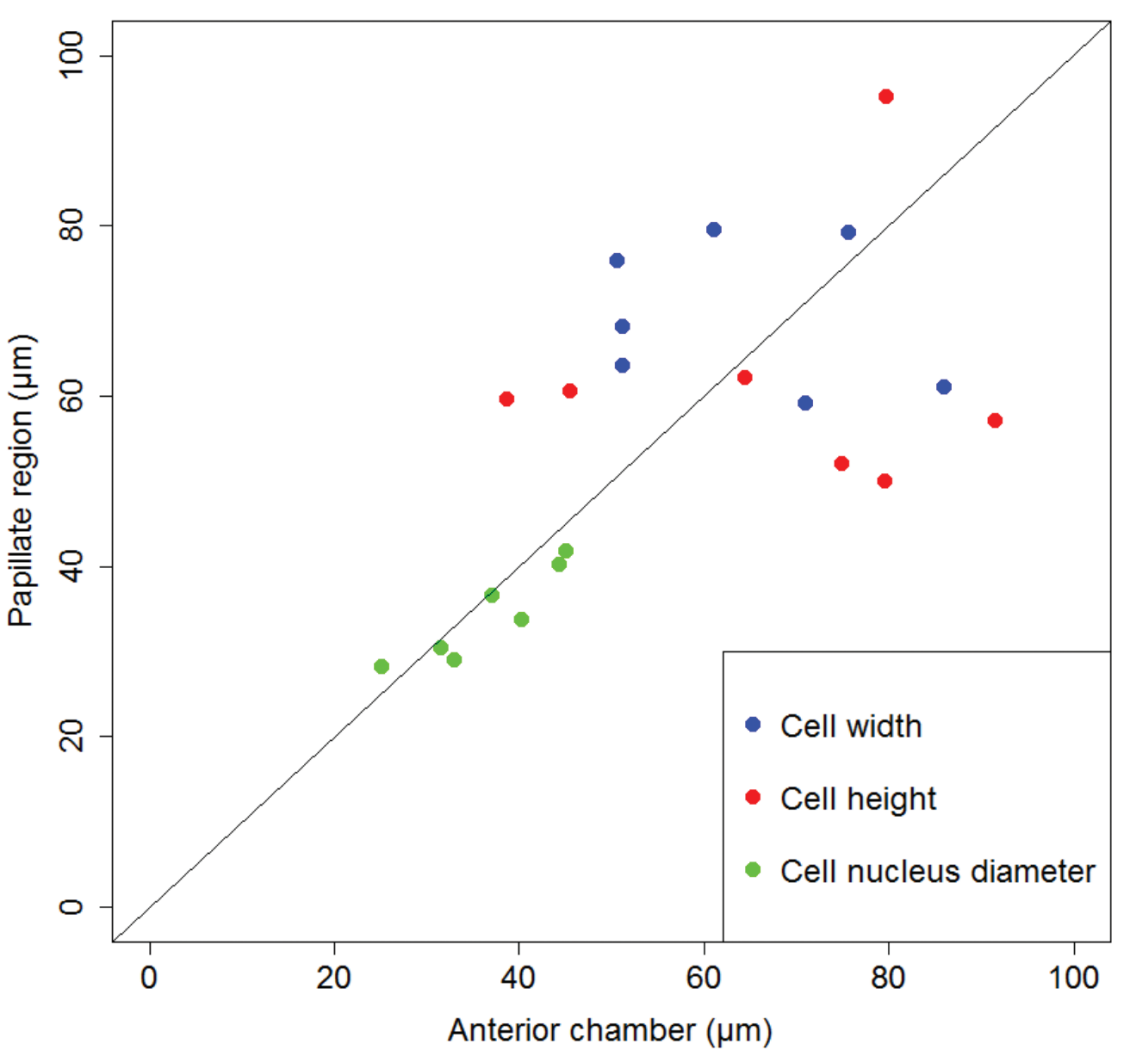
Cell width, cell height and cell nucleus diameter in the two hindgut regions. Scatter plot depicting median values of cell width, cell height and cell nucleus diameter in the anterior chamber against median values of same parameters in the papillate region of seven specimens. The central line is the line of equality. Points lying on the line indicate that the median value of measured parameter is equal in both hindgut regions. Points below the line indicate that the median value of measured parameter is larger in the anterior chamber than in the papillate region. Points above the line indicate that the median value of measured parameter is larger in the papillate region than in the anterior chamber.

Our measurements of cell width, cell height and cell nuclei size do not reveal difference in the size of the cells and their nuclei between the two hindgut regions (Fig. 3). Cells in both regions are large and the majority of the cells in the anterior chamber are 40 to 80 µm wide and 40 to 100 µm high. In the papillate region the majority of the cells are 60 to 90 µm wide and 50 to 80 µm high. All hindgut cells have large cell nuclei, which in a majority of the cells are between 20 and 50 µm in diameter (Suppl. material 1).

### Cuticle ultrastructure

**Figure 4.**
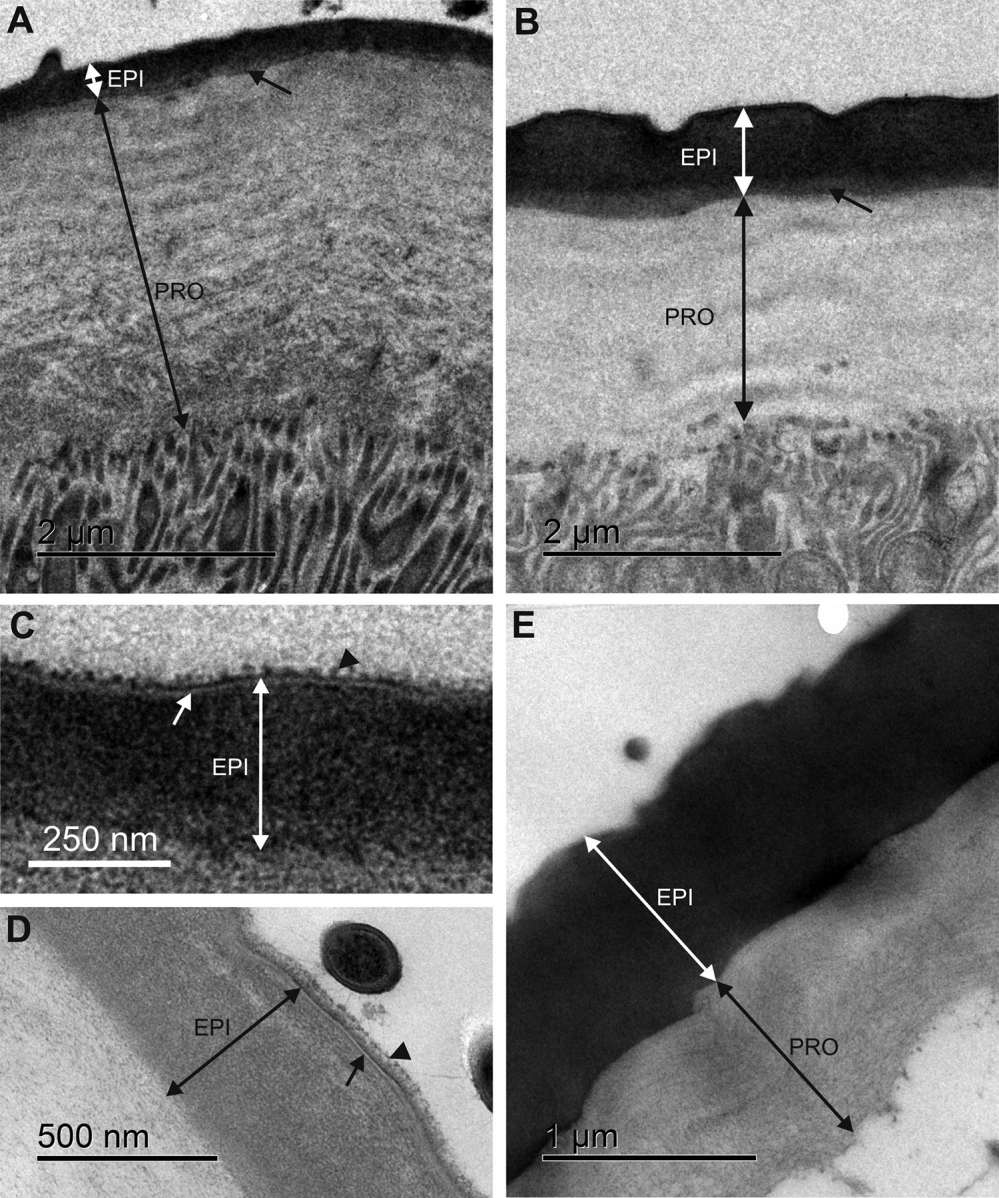
Ultrastructure of the cuticle in the anterior chamber and the papillate region. **A** The cuticle in the anterior chamber consists of thin epicuticle (EPI) and thick procuticle (PRO) with distinct sublayers. Between the epicuticule and the procuticle, a thin layer of intermediate electron density is present (arrow) **B** Cuticle in the papillate region consists of epicuticle (EPI) which is proportionally thicker according to the procuticle (PRO). Procuticle sublayers are less pronounced than in the anterior chamber. Between the epicuticle and the procuticle a thin layer of intermediate electron density is present (arrow) **C, D** The outer part of the epicuticle (EPI) in the anterior chamber (**C**) and in the papillate region (**D**) is three-layered (arrow) and covered with a fuzzy layer (arrowhead) **E** Procuticle (PRO) in the papillate region can be of approximately the same thickness as epicuticle (EPI) and without apparent sublayers.

**Figure 5.**
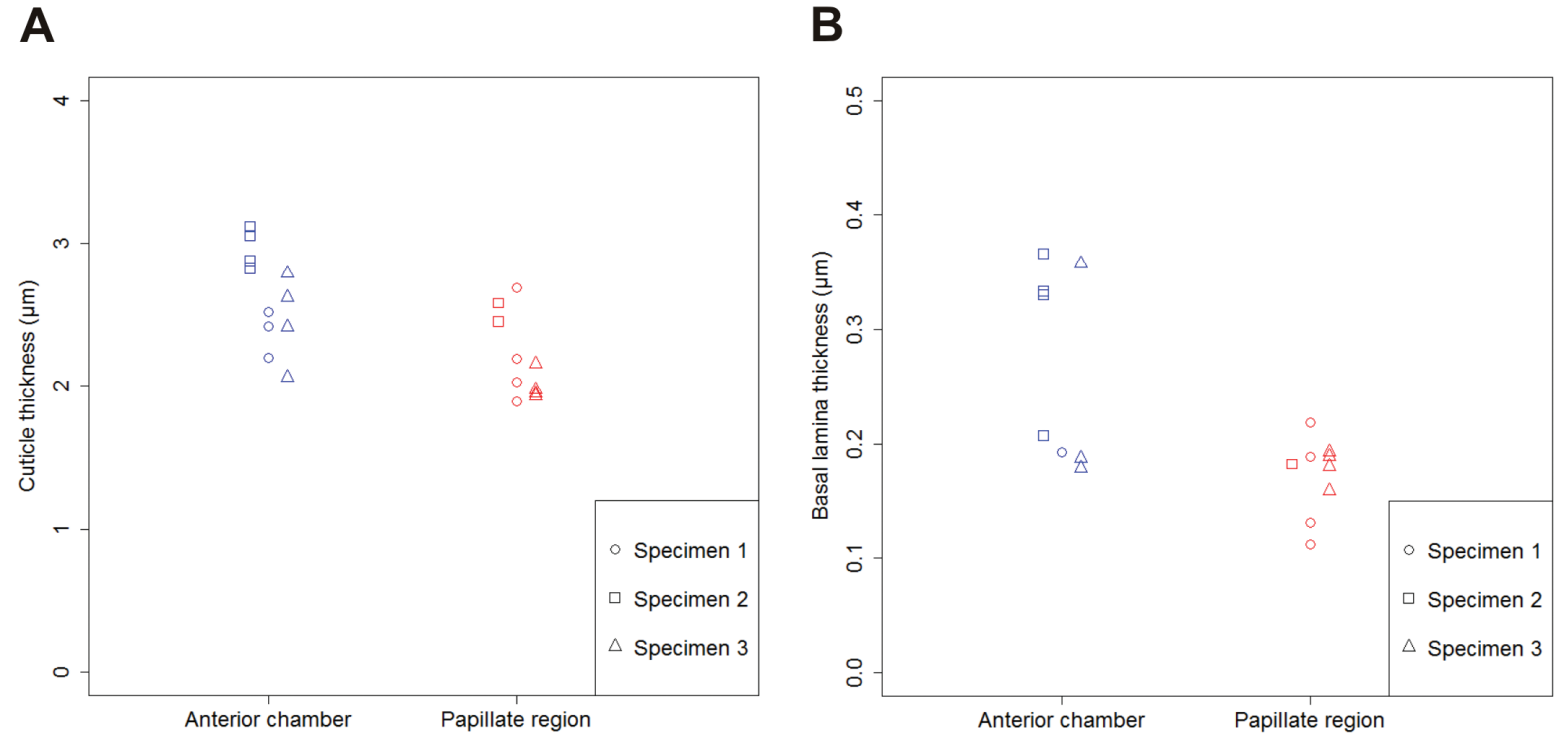
Cuticle and basal lamina thickness in the two hindgut regions. Median values of cuticle thickness (**A**) and basal lamina thickness (**B**) in cells from the anterior chamber (blue) and the papillate region (red) of three specimens.

**Figure 6.**
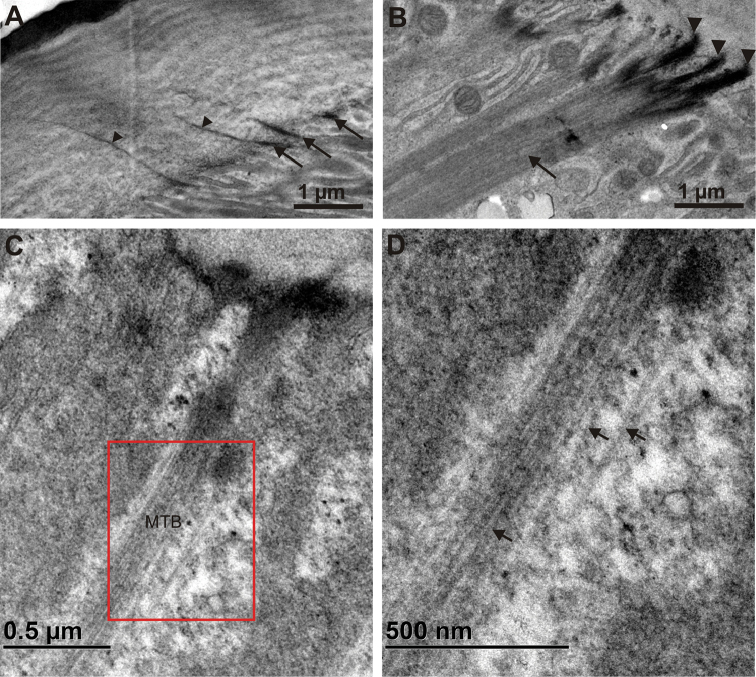
Ultrastructure of junctions between cuticle and apical plasma membrane. **A** Junctions between cuticle and apical plasma membrane in the anterior chamber visible as electron dense structures (arrows), from which fibers (arrowheads) extend into the procuticle **B** Junctions between cuticle and apical plasma membrane in the papillate region (arrowheads) associated with abundant bundles of microtubules inside the cell (arrow) **C, D** Higher resolution images of bundles of microtubules (MTB) associated with junctions between cuticle and apical plasma membrane. Image **D** displays the area denoted on image **C** (red rectangle) where individual microtubules (arrows) can be discerned.

The apical side of the epithelium in both hindgut regions is lined by a cuticle, which consists of an outer electron dense epicuticle and an inner electron lucent procuticle (Fig. 4A, B). The epicuticle consists of thin three-layered outer part and thicker electron dense inner part. The outer part of epicuticle is about 10 nm thick and consists of two electron dense sheets enclosing a central electron lucent sheet. The inner part of epicuticle is homogenous and 300–500 nm thick in the anterior chamber and 700–900 nm thick in the papillate region. The outer part of epicuticle is covered with a fuzzy layer on the luminal surface (Fig. 4C, D). Between the epicuticle and the procuticle a thin layer of intermediate electron density is present (Fig. 4A, B). No pore canals were observed in the hindgut cuticle. The thickness of the entire cuticle in both hindgut regions is in the range between 1.5 and 3 µm (Fig. 5A, Suppl. material 2). A notable difference exists in the ratio of epicuticle to procuticle thickness. In the anterior chamber the procuticle is significantly thicker than the epicuticle and the ratio of epicuticle to procuticle thickness is approximately 1:5 (Fig. 4A). The ratio of epicuticle to procuticle thickness in the papillate region is between 1:3 and 1:1 (Fig. 4B, E). In the papillate region the epicuticle is thicker than in the anterior chamber. The difference was also observed in the procuticle sublayers that appear due to helicoidally arranged chitin protein fibres. In the anterior chamber the procuticle sublayers can be distinguished (Fig. 4A). The procuticle sublayers in the papillate region are either less pronounced and thicker than in the anterior chamber (Fig. 4B) or not visible (Fig. 4E). In addition, the ultrastructure of connections between apical plasma membrane and cuticle is shown. Connections are present in individual epithelial cells in both hindgut regions and consist of electron dense fibres, which extend from the electron dense plaques at the apical cell surface into the procuticle. These fibres are more prominent in the anterior chamber (Fig. 6A). Connections in both hindgut regions are associated with apico-basally oriented bundles of microtubule-like filaments. These bundles are more abundant in the papillate region (Fig. 6B, C, D).

**Figure 7.**
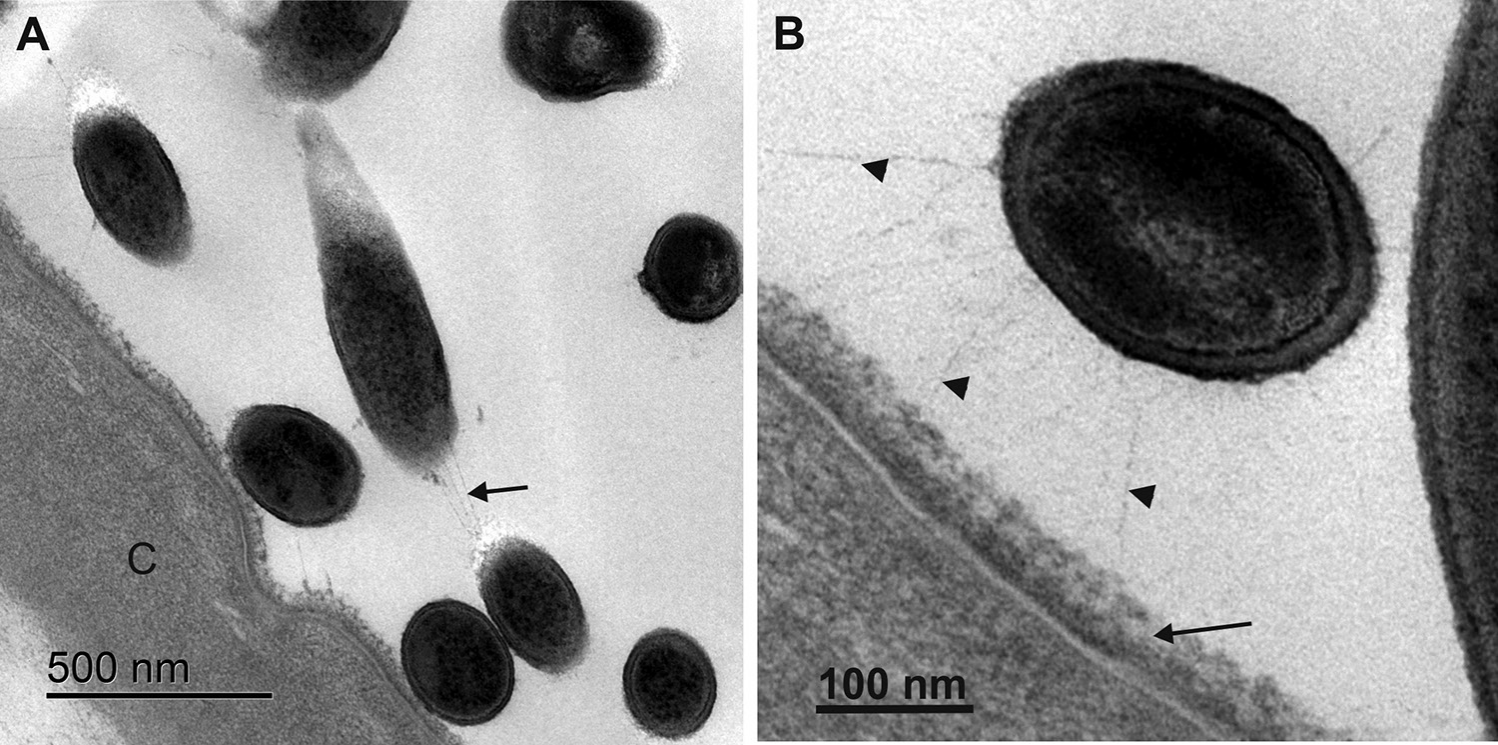
Bacteria associated with hindgut cuticle in the papillate region of one specimen. **A** bacteria are visible near or at the cuticle (C) surface. Bacterial cells are connected to each other with thin filamentous structures (arrow) **B** bacteria are connected to the fuzzy layer of epicuticle (arrow) by thin filamentous structures (arrowheads).

In the papillate region of one specimen, bacteria were observed near the cuticle or directly attached to the fuzzy layer at the cuticle surface (Fig. 7A). Spherical or rod-shaped profiles of sectioned bacteria were noted. A dense cytoplasm is enveloped by two electron dense sheets enclosing the middle electron lucent sheet. bacteria are connected to the fuzzy layer of epicuticle by thin filamentous structures (Fig. 7B). The thin filamentous connections between bacterial cells are also clearly resolved (Fig. 7A).

### Apical and basal plasma membrane labyrinths

**Figure 8.**
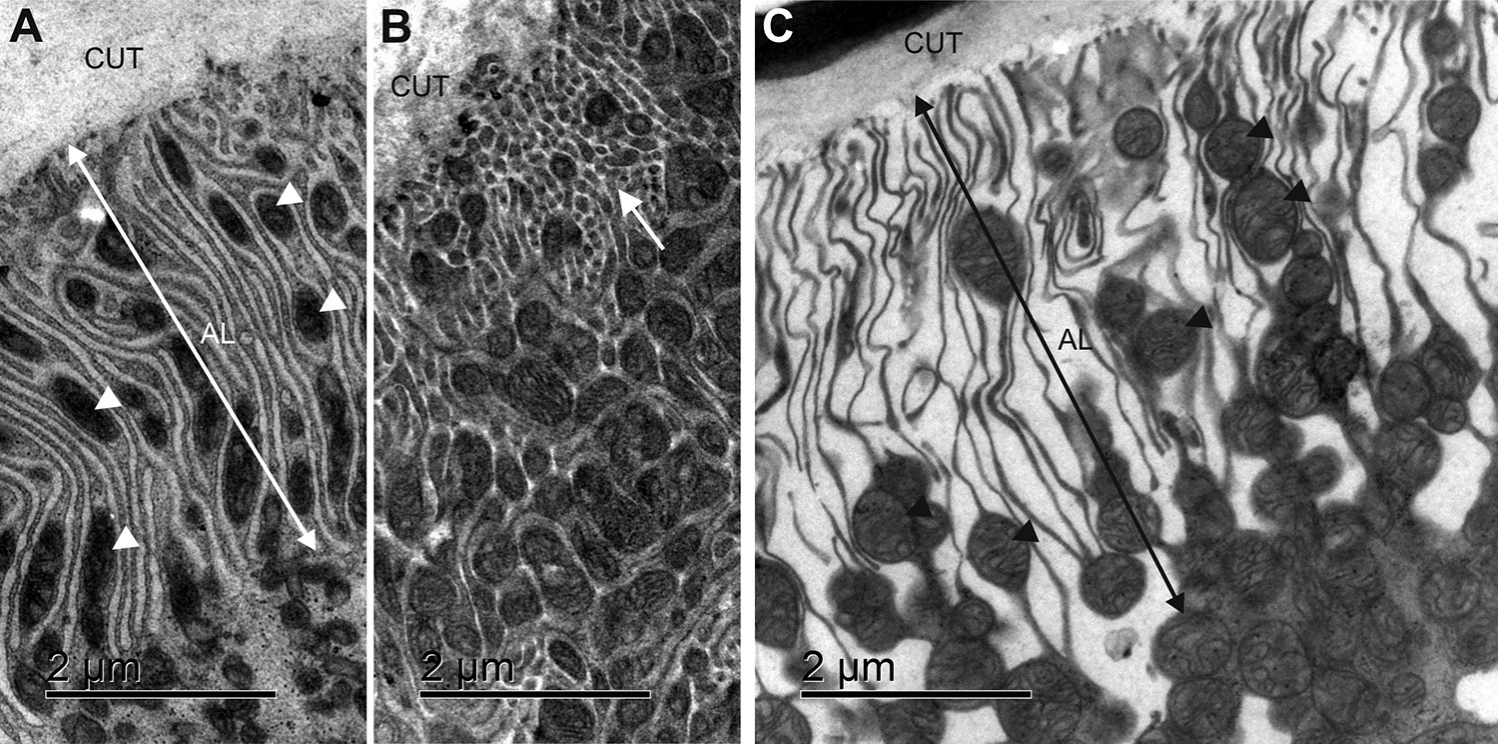
Ultrastructure of the apical plasma membrane labyrinth in the anterior chamber and the papillate region. **A** Apical membrane labyrinth (AL) in the anterior chamber. In the cytoplasmic strands mitochondria (arrowheads) are present **B** A tubular appearance of cytoplasmic strands is evident in certain sections (arrow) **C** Apical membrane labyrinth (AL) in the papillate region. Key: arrowheads – mitochondria, CUT – cuticle.

The general characteristic of the apical and basal plasma membranes of epithelial cells in both hindgut regions is that they are infolded, and form apical and basal plasma membrane labyrinths. However, considerable differences between the two hindgut regions were observed in the depth of the membrane infoldings, in the spatial density of membrane infoldings and in the abundance of mitochondria associated with membrane infoldings. The apical plasma membrane in cells of both hindgut regions forms extensive and complex labyrinth of cytoplasmic strands and extracellular spaces. Numerous mitochondria are present inside the cytoplasmic strands (Figs 8A, B, C). In the anterior chamber, the cytoplasmic strands are separated by extracellular spaces that are of approximately the same width as the cytoplasmic strands (Fig. 8A). Certain sections indicate that cytoplasmic strands are tubular structures rather than leaflets (Fig. 8B). In the papillate region the cytoplasmic strands are narrow and separated by wide extracellular spaces (Fig. 8C).

**Figure 9.**
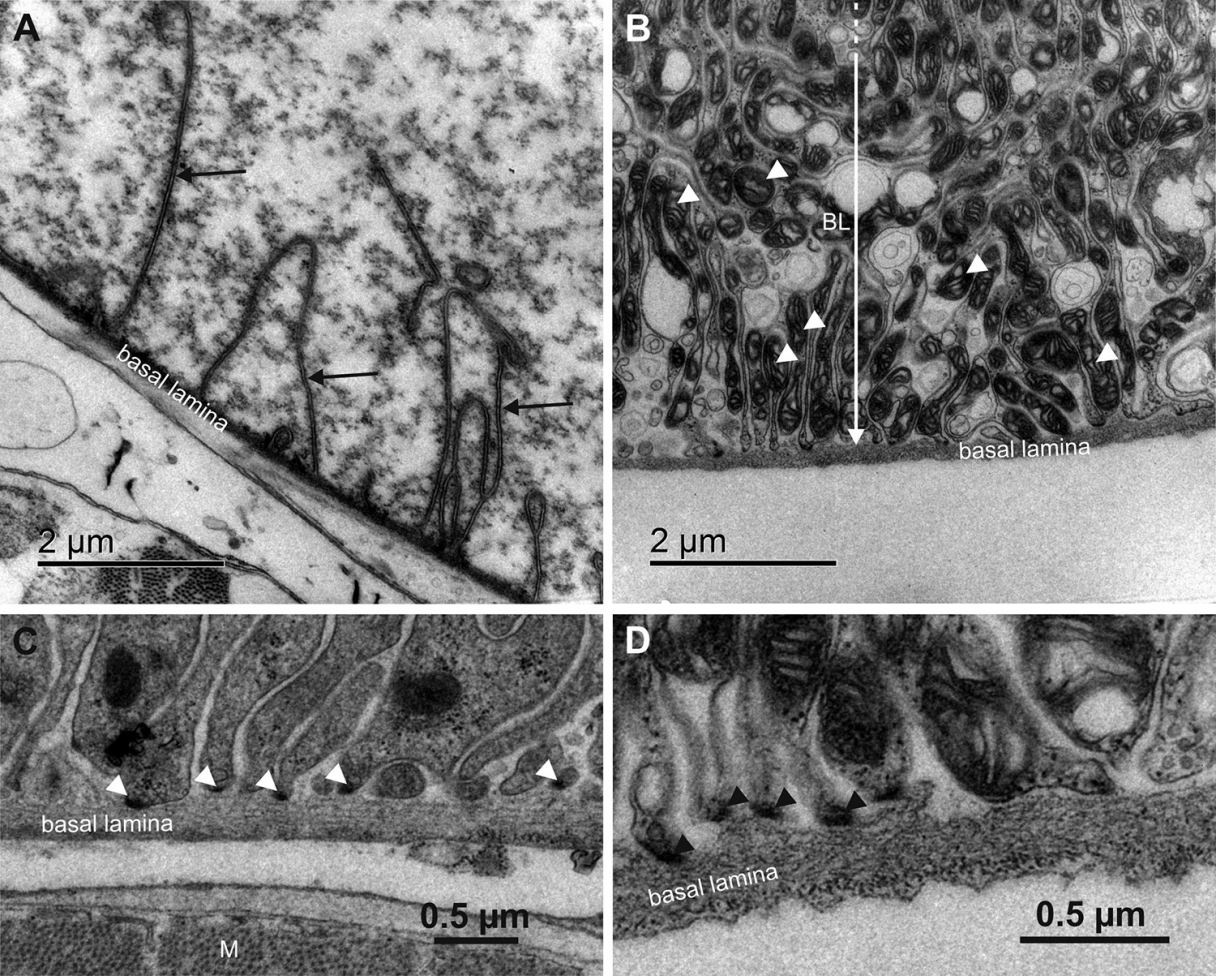
Ultrastructure of the basal plasma membrane labyrinth in the anterior chamber and the papillate region. **A** Basal plasma membrane in the anterior chamber forms sparse narrow infoldings (arrows) **B** Extensive basal membrane labyrinth (BL) in the papillate region is associated with numerous mitochondria (arrowheads) **C** Basal lamina in the anterior chamber. Hemidesmosome-like junctions are visible as small electron dense plaques (arrowheads) **D** Basal lamina in the papillate region. Hemidesmosome-like junctions are visible as small electron dense plaques (arrowheads). Abbreviation: M – muscle.

The differences in the basal membrane labyrinths of epithelial cells in the two hindgut regions are even more prominent. The basal plasma membrane in the anterior chamber forms relatively sparse narrow infoldings which are accompanied by scant mitochondria (Fig. 9A). The abundance of mitochondria in the vicinity of basal infoldings is no greater than elsewhere in the cytoplasm. In the papillate region the infoldings of basal plasma membrane are closely spaced and form a deep basal labyrinth. Mitochondria in the papillate region are concentrated in the area of basal labyrinth and associate with the basal membrane infoldings (Fig. 9B). In both hindgut regions the basal plasma membrane is supported by an outstandingly thick basal lamina, measuring typically between 100 and 300 nm. In the anterior chamber in particular, the basal lamina can reach up to 400 nm in thickness (Fig. 5B, Suppl. material 3). Hemidesmosome-like junctions, visible as small electron dense plaques (Fig. 9C, D), were observed at sites where the basal plasma membrane is in contact with the basal lamina.

**Figure 10.**
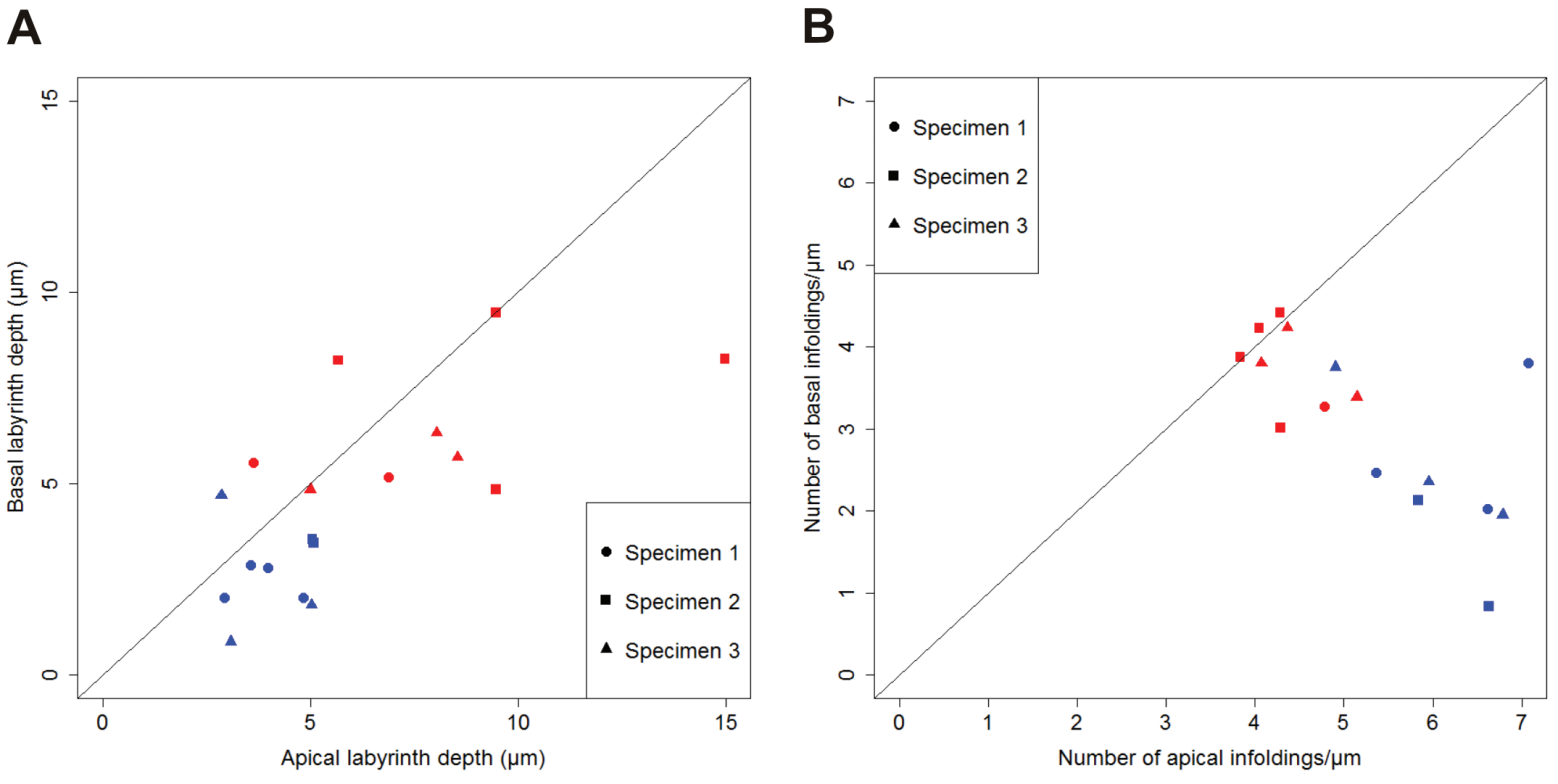
Apical and basal labyrinth depth and density of membrane infoldings in the two hindgut regions. Scatter plots depict: **A** Median values of apical labyrinth depth versus the median values of basal labyrinth depth in cells from the anterior chamber (blue) and the papillate region (red) of three specimens **B** Median values of apical infoldings density against the median values of basal infoldings density in cells from the anterior chamber (blue) and the papillate region (red) of three specimens. The central line in both plots is the line of equality. Points lying on the line indicate that median value of depth/density is equal, apically and basally. Points below the line indicate that the median value of depth/density is larger apically than basally. Points above the line indicate that the median value is larger basally than apically.

Measurements of the membrane labyrinths depth suggest that the apical and the basal labyrinths are both deeper in the papillate region in comparison to the anterior chamber and that in both hindgut regions the apical membrane labyrinth is slightly deeper than the basal membrane labyrinth (Fig. 10A). The apical labyrinth is mainly between 2 and 5 µm deep in the anterior chamber and between 3 and 15 µm deep in the papillate region (Suppl. material 4). The basal labyrinth is mainly between 1 and 4 µm deep in the anterior chamber and between 3 and 10 µm deep in the papillate region (Suppl. material 5). Measurements of the spatial density of membrane infoldings suggest that in the anterior chamber the apical infoldings are more closely spaced than the basal infoldings, while in the papillate region the spatial densities of apical and basal infoldings are more equal (Fig. 10B). The apical membrane infoldings are more closely spaced in the anterior chamber (5–8 infoldings/µm) than in the papillate region (3–6 infoldings/µm) (Suppl. material 6). The basal membrane infoldings are more closely spaced in the papillate region (3–5 infoldings/µm) than in the anterior chamber (1–4 infoldings/µm) (Suppl. material 7).

### Cell junctions

**Figure 11.**
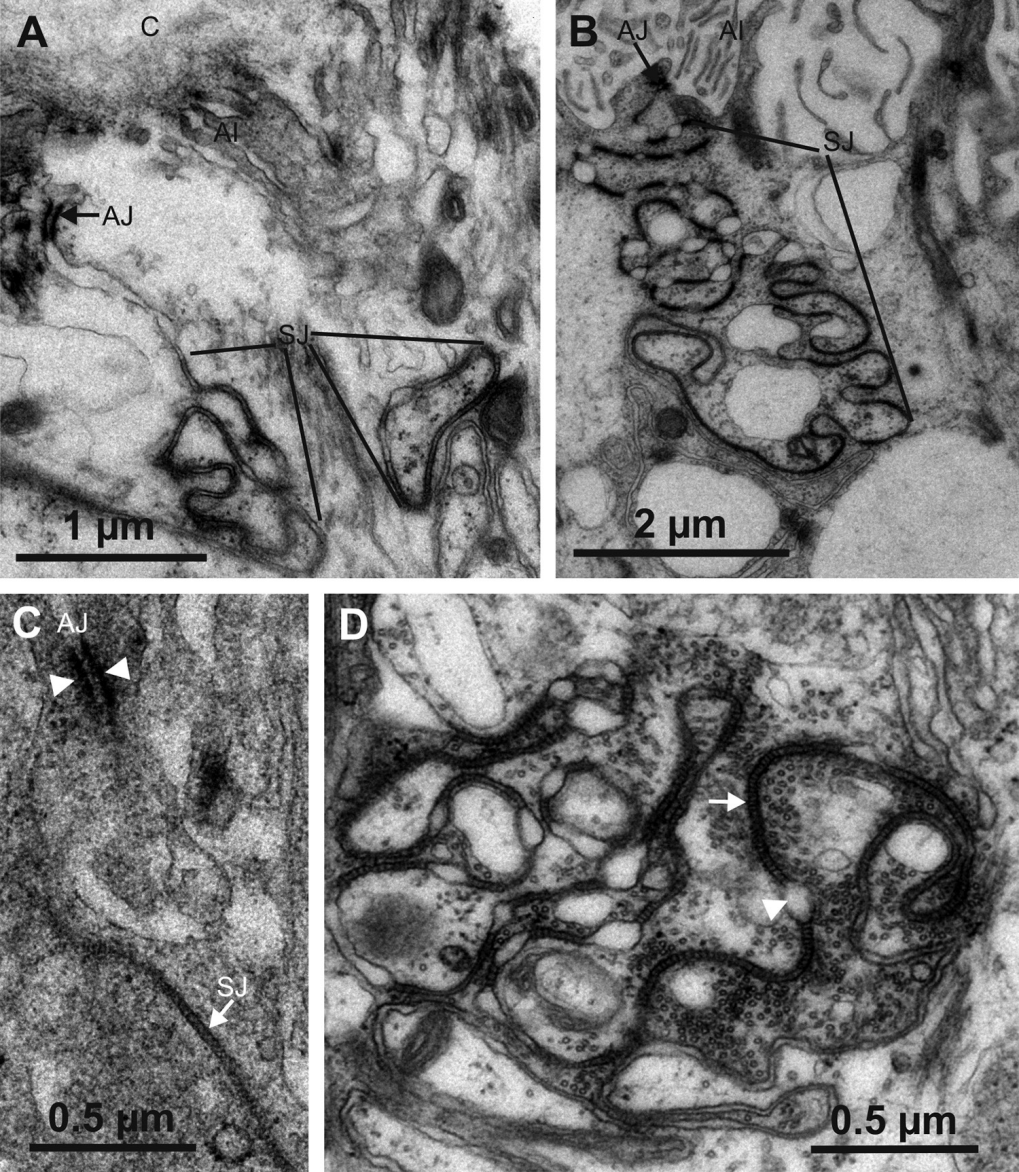
Ultrastructure of cell junctions in the anterior chamber and the papillate region. **A** The junctional complex in the anterior chamber consists of subapically located adherens junctions (AJ) and septate junctions (SJ) located beneath the adherens junctions **B** The junctional complex in the papillate region consists of subapically located adherens junctions (AJ) and extremely long and convoluted septate junctions (SJ) located beneath the adherens junctions **C** Adherens junctions consist of two electron dense plaques (arrowheads) at the cytoplasmic sides of lateral plasma membranes of two neighbouring cells and electron dense material in the intercellular space between the membranes **D** Septate junctions are visible as electron dense septa arranged in strings (arrow). Dilated intercellular spaces are visible where septate junctions are locally interrupted (arrowhead). Abbreviations: C – cuticle, AI – apical infoldings, SJ – septate junction.

The junctional complexes between epithelial cells in both hindgut regions consist of subapically located adherens junctions and septate junctions which are located beneath the adherens junctions (Fig. 11A, B). Ultrastructurally, the adherens junctions appear to be alike in both hindgut regions. They consist of two electron dense plaques at the cytoplasmic sides of lateral plasma membranes of the two neighbouring cells and electron dense material in the intercellular space between the membranes (Fig. 11C). The ladder-like septate junctions are visible as strings of electron dense septa in the intercellular space between lateral plasma membranes of the two neighbouring cells. Strings of electron dense septa are interrupted at some sites by dilated intercellular spaces. Abundant microtubules are present in the vicinity of septate junctions (Fig. 11D).

**Figure 12.**
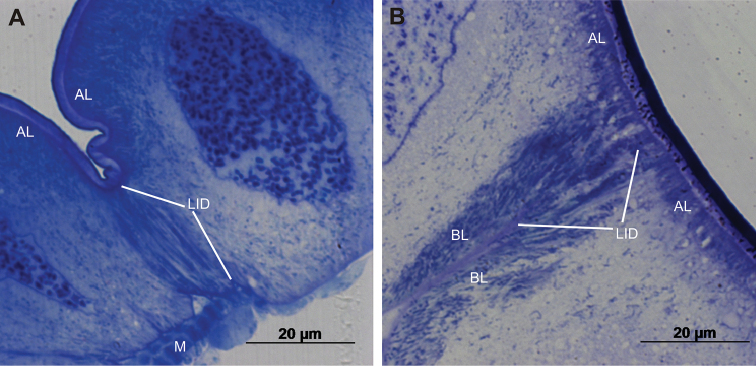
Lateral parts of epithelial cells in the anterior chamber and the papillate region. **A** Two neighbouring cells in the anterior chamber **B** Two neighbouring cells in the papillate region. Abbreviations: LID – area of lateral interdigitations, AL – apical labyrinth, BL – basal labyrinth, M – muscle.

Septate junctions in both hindgut regions occupy considerable portions of the lateral membranes. In the papillate region the membrane area with septate junctions is intensely convoluted and the overall length of septate junctions is larger than that of the anterior chamber (Fig. 11). Large parts of lateral plasma membranes, which lie basally of the septate junctions, are heavily interdigitated between neighbouring cells (Fig. 12).

### Na^+^/K^+^-ATPase activity localisation

**Figure 13.**
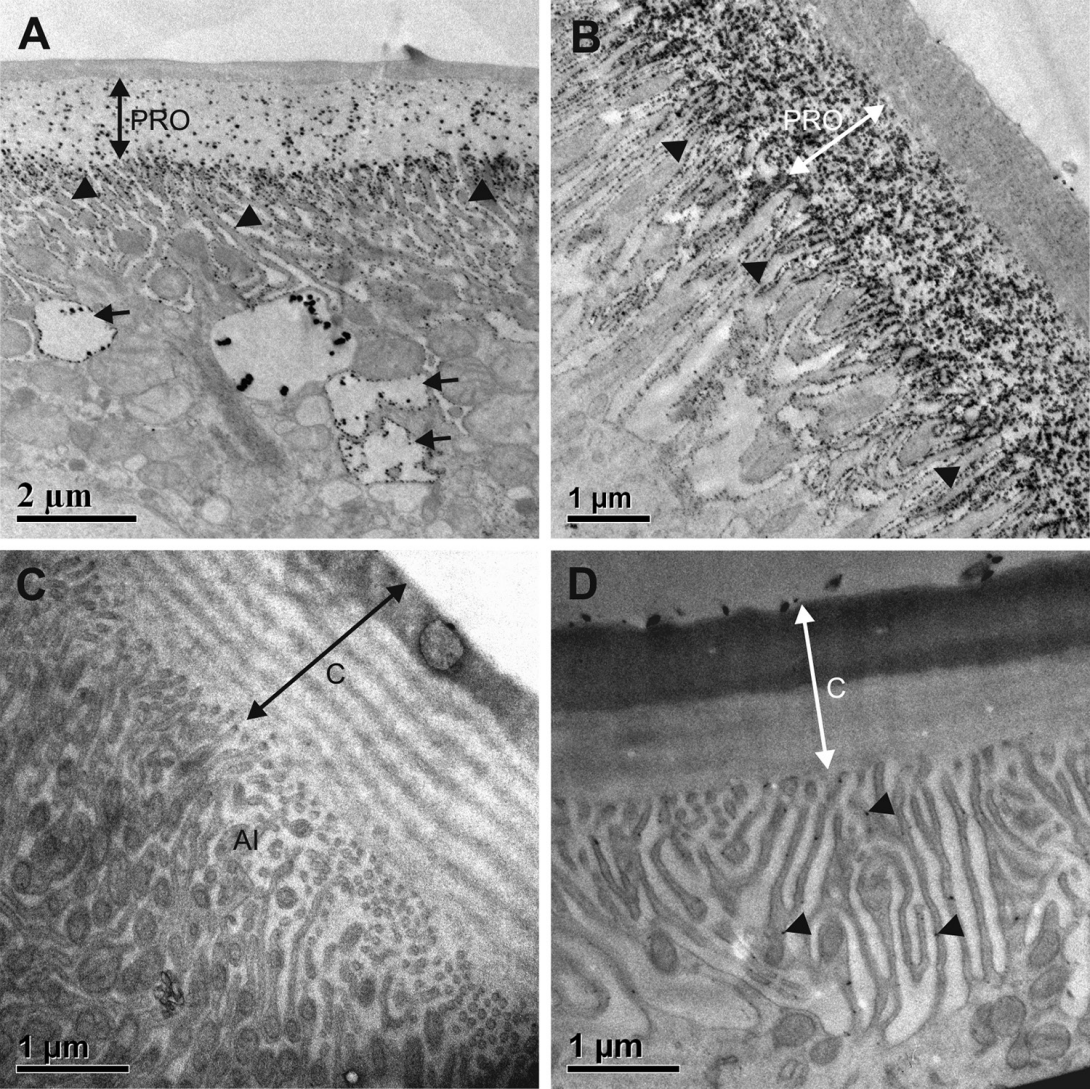
Localization of Na^+^/K^+^-ATPase activity in hindguts of intermoult and postmoult animals. **A** Na^+^/K^+^-ATPase activity in the anterior chamber of intermoult *P.scaber*. Electron dense deposits are present along tubular (arrowheads) and dilated (arrows) apical membranous infoldings and in the procuticle (PRO) **B** Na^+^/K^+^-ATPase activity in the papillate region of postmoult *P.scaber*. Abundant deposits are present along the apical infoldings (arrowheads) and in the procuticle (PRO) **C** Control section in the anterior chamber of intermoult animal where K^+^ ions were replaced by Na^+^ ions. No reaction product is present along apical infoldings (AI) or in the cuticle (C) **D** Control section in the papillate region of postmoult animal treated with ouabain. Some deposits (arrowheads) are present along the apical infoldings. Abbreviations: C – cuticle.

Na^+^/K^+^-ATPase activity was localised indirectly through lead phosphate deposits, which were present in the procuticle and along apical membranous invaginations of the anterior hindgut in postmoult and intermoult animals. The deposits were observed in tubular and dilated vacuolar infoldings (Fig. 13A) and were not localised in apical mitochondria. In the hindgut papillate region Na^+^/K^+^-ATPase activity was very intense in postmoult animals (Fig. 13B) with abundant reaction product in newly secreted procuticle and along deep tubular apical infoldings. Some reaction product was present also along basal membrane infoldings. In control sections where K^+^ ions were replaced by Na^+^ ions, deposits were not detected (Fig. 13C), but some deposits were present in controls treated with ouabain (Fig. 13D).

## Discussion

The present study complements and upgrades the previous knowledge of the hindgut epithelial cell ultrastructure in isopods by providing a detailed description and quantification of ultrastructural characters of these cells and their comparison between the main two hindgut regions. We also report on connections between the epithelial cells and the hindgut cuticle, which were not described in previous works. Our results show clear evidence of prominent ultrastructural differences between the epithelia in the anterior chamber and in the papillate region. Cuticle structure and the thickness of epicuticle and procuticle differ significantly between the two hindgut regions, which suggests different mechanical and functional aspects of the cuticle, such as permeability, stiffness, and compactness. Another feature that differentiates epithelial cells in the two hindgut regions is the intensity of apical and basal membrane infolding. This is likely related to transport of nutrients, water and ions through epithelia between the hindgut lumen and haemocoel. Epithelial cells differ also in the extent of septate junctions and the interdigitations of lateral plasma membranes, which are important for the epithelial paracellular permeability in the two hindgut regions.

Epithelial cells in the hindgut of *P.scaber* are in general columnar to isodiametric and large, measuring between 40 and 100 µm in diameter. We consider that the cell width and height varies also due to contractions of an extensive neuromuscular network and due to distension of epithelium in guts filled with food. Measured epithelial cells in the papillate region were flatter since the papillate region in majority of specimens was completely full and epithelium was more distended.

Both the hindgut epithelium and the epidermis in crustaceans are of ectodermal origin and are apically lined by a chitinous cuticle. A structural comparison between these two different cuticles in the same species is helpful in the evaluation of the observed cuticle characteristics in relation to their specific functions. While the epidermis with the mineralised exoskeleton provides protection against predators, infections and desiccation, communicates with external environment and provides mechanical support of the body crucial for locomotion, the hindgut epithelium is involved in transport of nutrients, water and ions (Hryniewiecka-Szyfter and Storch 1986, Hames and Hopkin 1989). The ultrastructure and composition of exoskeletal cuticle in isopods, including *P.scaber*, have been described precisely (Compere 1991, Štrus and Compere 1996, Ziegler 1997a, Štrus and Blejec 2001, Hild et al. 2008, 2009, Seidl et al. 2011, Seidl and Ziegler 2012, Vittori and Štrus 2014) and here the ultrastructure of the *P.scaber* hindgut cuticle is discussed in respect to the exoskeleton of this animal. The hindgut cuticle of *P.scaber* is about ten times thinner than the exoskeletal cuticle, which is likely a modification for passage of molecules and ions. But on the other hand, the hindgut epicuticle is between one and four-times thicker than that of the exoskeleton (~150 to 250 nm). Accordingly, the ratio of epicuticle thickness to procuticle thickness is considerably higher in hindgut cuticle than in exoskeletal cuticle of *P.scaber*. The hindgut cuticle is connected to individual epithelial cells by cuticle–epithelium attachments, which ultrastructurally resemble connections between the exoskeletal cuticle and specialised epidermal cells (tendon cells) in arthropods (Buchholz and Buchholz 1989, Mellon 1992, Lai-Fook and Beaton 1998, Bitsch and Bitsch 2002, Žnidaršič et al. 2012).

The hindgut epicuticle of *P.scaber* structurally resembles the homogenous inner and trilayered outer epicuticle of the animals’ exoskeleton. The detailed description of the outer epicuticle in isopod exoskeletons revealed that it usually consists of several thin layers (Ziegler 1997a, Compere 1991, Vittori and Štrus 2014). The additional electron dense layer, deposited inside the outer epicuticle of exoskeleton, has been referred to as the ‘waxy layer’ and is presumably composed of lipids and acts as a surface waterproofing barrier (Compere 1991). This layer was not distinguished in *P.scaber* hindgut epicuticle and thus may not be present in this case. This could imply better permeability of the hindgut cuticle. The fuzzy layer covering the epicuticle surface has already been reported in *P.scaber* hindgut by Holdich and Mayes (1975) as a diffuse outer layer of epicuticle. It resembles the exoskeletal ‘surface coat’, which has been reported to be more pronounced in aquatic crustaceans (Compere 1995) and thinner in isopods (Compere 1991, Vittori and Štrus 2014). The function and composition of the surface coat in the crustacean exoskeleton is not fully understood. More data are available for the similar fuzzy surface coat overlying the trilayered epicuticle in nematode integument, but it must be stressed here that the nematode cuticle is composed largely of collagen and is in this concern not directly comparable to the chitinous cuticle of arthropods. The main constituents of the nematode surface coat appear to be glycoproteins and proteins and its biological role is mainly assigned to the interactions between nematodes and microorganisms, as well as between parasitic nematodes and their hosts (Jansson et al. 1986, Wright 1987, Blaxter et al. 1992, Spiegel and McClure 1995). In the papillate region of one specimen we observed bacteria attached to the fuzzy layer of epicuticle with thin filamentous structures. In terrestrial isopods it is known that some bacteria are able to attach to the hindgut cuticle. This has been reported for the rod-like bacteria *Candidatus* Bacilloplasma, which is known to attach to the tips of cuticular spines in the papillate region of *P.scaber* with spherical attachment structures (Kostanjšek et al. 2003, Kostanjšek et al. 2006, Kostanjšek et al. 2007). The inner epicuticle is thicker in the papillate region than in the anterior chamber. This may influence different epithelial permeability and mechanical properties. Zimmer (1999) reported that the cuticle in the anterior chamber of *P.scaber* is permeable to the molecules up to the size of the *tri*-galloyl-glucose, while in the papillate region the cuticle is impermeable for the molecules of the size of glucose. The cuticle in the anterior chamber thus allows the absorption of digestive products up to the size of 1.9 nm (Zimmer 2002). Thin vertical canals in the inner epicuticle described in insect and crustacean exoskeletons (Locke 1961, Compere and Goffinet 1987a, b, Compere 1991, 1995, Ziegler 1997a), were not observed in our study of the hindgut cuticle. Epicuticular canals in exoskeleton serve as access routes for lipids in insects (Locke 1961) and it has been suggested that they may be related to the waxy layer formation in the isopod exoskeleton (Compere 1991).

The procuticle in the hindgut displays distinctive sublayers, which are more obvious in the anterior chamber. The hindgut cuticle has thinner procuticle relative to the entire cuticle thickness, and structurally displays less pronounced organisation of the chitin-protein lamellae than the exoskeletal cuticle. The lamellar organisation contributes to exoskeletal cuticle stiffness and mechanical resistance as a consequence of the complex hierarchical structure at all levels of organisation, from molecules to helicoidally stacked chitin-protein planes (Raabe et al. 2005, 2006, Fabritius et al. 2009, Dillaman et al. 2013). The exoskeletal cuticle is in addition hardened by mineralisation (Compere et al. 2004, Luquet 2012, Dillaman et al. 2013). The difference between the hindgut and exoskeletal procuticles suggests different mechanical properties and indicates lower stiffness of the hindgut cuticle in comparison to that of the exoskeleton. As it is proposed for decapod exoskeleton, a higher stacking density of the chitin-protein planes leads to greater cuticle stiffness and hardness (Raabe et al. 2005). In this regard, a closer resemblance of the procuticle in the anterior chamber to that of the exoskeleton, with more numerous and thinner lamellae, implies higher stiffness of the cuticle in this region than in the papillate region. This is in favour of active digestion of coarse food particles and intensive fluxes in relation to backflow of digested material through typhlosolis channels in the anterior chamber. These processes are caused by mechanical compression of the gut content performed by the surrounding muscular network, which is more pronounced in this region than in the papillate region. Pore canals traversing the cuticular matrix, generally characteristic of the exoskeletal cuticle, were not observed in the hindgut cuticle. Absence of pore canals is also characteristic of some flexible and elastic cuticles, such as cuticles of locust wing hinges and the pleural arch of fleas (Neville 1975), both of which contain the elastic protein resilin (Weis-Fogh 1960, Burrows et al. 2008). Structurally similar procuticle as in the *P.scaber* papillate region, i.e. without discernible division between exocuticle and endocuticle and no clearly distinct sublayers, is present in cuticles of some other transporting epithelia of arthropods. In the marine isopod *Jaeranordmanni*, the cuticle of ion transporting gill epithelium consists of epicuticle and amorphous flocculent material underneath (Bubel and Jones 1974). The very thin cuticle of gills in dragonfly nymphs consists of epicuticle and procuticle in up to two sublayers (Neville 1975). The tracheal cuticle of insects also consists of epicuticle and conspicuous procuticle with amorphous organisation of chitin (Moussian 2013). Cuticles of ion transporting epithelia often display epicuticular depressions as in the rectal chloride epithelium of larval dragonflies (Kukulies and Komnick 1983) and the rectal papillae of *Muscadomestica* (Flower and Walker 1979) or porous plates as in mayfly larval gills (Komnick and Stockem 1973, Neville 1975). Pores of different diameters have been reported at the cuticle surface in the hindgut of locust *Locustamigratoria*. Smaller pores in the cuticle covering the rectal pads are probably involved in water resorption (Klein and Applebaum 1975). We did not observe any such specialisations in the hindgut cuticle of *P.scaber*. Overall, our results suggest that the hindgut cuticle of *P.scaber* shares some structural characteristics with some other cuticles in transporting epithelia and with some cuticles displaying a pronounced elasticity.

Infolded plasma membranes associated with mitochondria, as reported here for the hindgut epithelium, are a general characteristic of transporting epithelial cells. The infolded apical, lateral or basal plasma membranes enlarge the surface across which the transport can take place and provide enclosed extracellular spaces, where ion gradients can build up. This in turn can drive secondary transport and the flow of water. Associated mitochondria are important in providing energy for the active transport of ions (Cioffi 1984, Fukudome 2001, Weihrauch and O'Donnell 2015). The differences in the extent of apical and basal surface amplification and in the association of mitochondria with plasma membrane infoldings indicate differences in function. In the anterior chamber the apical surface of epithelial cells is amplified and associated with mitochondria in particular. This reflects intensive material exchange between epithelial cells and the hindgut lumen, which supports the proposed role of anterior chamber in the absorption of nutrients (Hryniewiecka-Szyfter and Storch 1986, Hames and Hopkin 1989). In the papillate region both the apical and the basal surfaces of epithelial cells are greatly amplified and associated with mitochondria. This indicates intensive material exchange with both the hindgut lumen and the haemocoel, which is consistent with transepithelial transport of ions and water in the papillate region (Vernon et al. 1974, Coruzzi et al. 1982, Palackal et al. 1984). Transepithelial transport of ions and water in the papillate region can drive osmoregulatory processes, such as ion sequestration in the hindgut during dehydration (Wright et al. 1997) or salt excretion with faeces in supralittoral and littoral genera *Ligia* and *Tylos* (Wright et al. 2003).

Epithelial cells in the anterior chamber of *P.scaber* have their apical surfaces amplified by apical membrane infoldings. More commonly, the absorptive cells in digestive systems of arthropods are of endodermal origin and have apical surfaces amplified by brush border of microvilli (Cioffi 1984, Icely and Nott 1992, Vogt 1994, Shanbhag and Tripathi 2009). In terrestrial isopods, which lack endodermal midgut, such cells are present in hepatopancreas and probably fulfil the main part of nutrient absorption (Hames and Hopkin 1989). Epithelial cells in the papillate region of *P.scaber* are structurally similar to various epithelial cells involved in ion transport. Ion transporting cells in arthropods often have amplified apical membrane in the form of leaflets (Cioffi 1984). Examples of ion transporting epithelial cells with extensively infolded apical membranes are cells in the rectal papillae of *Calliphora* (Berridge and Gupta 1967), the salt transporting cells in crustacean gills (Bubel and Jones 1974, Taylor and Taylor 1992, Kikuchi and Matsumasa 1993, Luquet et al. 2000, Luquet et al. 2002) and distal duct cells in salivary glands of cockroach *Periplanetaamericana* which modify the primary saliva by absorption of Na^+^ ions and secretion of K^+^ ions (Just and Walz 1994). Besides the apical infoldings, extensive basal labyrinths associated with numerous mitochondria are also common in the salt transporting epithelial cells in crustacean gills and the distal duct cells in cockroach salivary glands. In the epidermis of *P.scaber* the anterior sternal epithelium is involved in Ca^2+^ transepithelial transport during premoult and intramoult, when the formation and resorption of sternal CaCO_3_ deposits take place. Anterior sternal epithelial cells in premoult and intramoult animals have amplified basolateral plasma membranes in the form of interstitial dilations and ramifying channels. In intramoult animals, the apical plasma membranes are also amplified by subcuticular folds. Such elaborations of basolateral and apical plasma membranes are absent in the posterior sternal epithelium (Ziegler 1996).

Two important ion pumps in ion transporting epithelia are the Na^+^/K^+^-ATPase and the vacuolar H^+^-ATPase (Weihrauch and O'Donnell 2015). In the hindgut of *P.scaber* Na^+^/K^+^-ATPase activity is localised primarily in the apical membranes of epithelial cells and scantly in the basal membranes. The intense ATPase activity in the apical membranous labyrinth of papillate region is probably due to extensive transepithelial transport of the water and ions necessary for osmoregulation. This apical localisation of Na^+^/K^+^-ATPase is rather unusual, since in general it is more common that the Na^+^/K^+^-ATPase is localised in the basolateral membranes of epithelial cells. In decapod Crustacea the Na^+^/K^+^-ATPase activity was cytochemically localised in the basolateral membranes of ion transporting cells in gill epithelia of crabs *Callinectessapidus* and *Carcinusmaenas* (Towle and Kays 1986). Na^+^/K^+^-ATPase was also immunolocalised in the basal membranes of branchiostegite epithelial cells in lobster *Homarusgammarus* (Lignot et al. 1999) and shrimp *Macrobrachiumamazonicum* (Boudour-Boucheker et al. 2014). Just and Walz (1994) immunolocalised Na^+^/K^+^-ATPase in the salivary gland of cockroach *P. Americana*. In this case ATPase is localised in the apical membranes in peripheral cells for secretion of NaCl rich primary saliva and in the basolateral membranes of distal duct cells which modify the saliva by absorption of Na^+^ and secretion of K^+^. In terrestrial isopods Na^+^/K^+^-ATPase activity was cytochemically localised in the basolateral or apical membrane infoldings in the posterior hindgut of *A.officinalis* (Warburg and Rosenberg 1989). Na^+^/K^+^-ATPase was also immunolocalised in the basolateral membranes of the anterior and posterior sternal epithelial cells of premoult and intramoult *P.scaber* (Ziegler 1997b).

The outstanding ultrastructural hallmarks of the hindgut epithelium are extensive septate junctions (SJ) located basal to adherens junctions. In different arthropod epithelia, two types of septate junctions have been described: pleated SJ and smooth SJ, which both typically form circumferential belts around the apicolateral regions of epithelial cells (Izumi and Furuse 2014, Jonusaite et al. 2016). Pleated SJ are generally observed in ectodermal epithelia, including epidermis, foregut and hindgut and their septa form regular undulating rows. Smooth SJ are present in endodermal epithelia and their septa are arranged in parallel lines. Data on morphology of arthropod SJ are extensive, but their functional aspects, molecular architecture and regulation are still not well characterised and these studies are limited mostly to *Drosophila*. Septate junctions are considered occluding junctions, restricting the passage of material through the paracellular pathway, but detailed properties of their barrier function are not clearly understood. The extensive SJ documented in our study imply that the paracellular transport is tightly regulated in the whole hindgut and in particular in the papillate region, where the SJ area is enlarged due to convoluted plasma membrane and where the cumulative number of septa spanning the intercellular space is higher. In the vicinity of septate junctions numerous microtubules were observed. As clusters of cross-sectioned microtubules were evident, we consider that these microtubules run parallel to lateral plasma membranes. A similar organisation of microtubules associated with septate junctions was observed in gill epithelium of some gammarid amphipods (Shires et al. 1995), which was interpreted as a protection against expansion and contraction of cells in the lateral plane necessary for the integrity of the permeability barrier.

The basal lamina beneath the basal plasma membrane of epithelial cells in both hindgut regions is outstandingly thick (100 – 300 nm). The typical thickness of basal laminae in vertebrates is between 50 and 100 µm (Ghadially 1997). Analyses of vertebrate basal laminae revealed their heterogeneity in molecular composition and organisation and elucidated diversity in their biological roles (LeBleu et al. 2007). Basal laminae fulfil many biological functions, among others they provide structural support for cells and serve as selective barriers. In recent years, valuable data on biomechanical properties of basal laminae became available, which importantly improved the understanding of their complex properties and dynamics (Halfter et al. 2015, Miller 2017). Data on basal laminae structure and properties in invertebrates are scarce. Reddy and Locke (1990) investigated the penetration of gold nanoparticles through different insect basal laminae. Basal laminae of tissues that synthesise and secrete haemolymph proteins, such as fat body, epidermis and pericardial cells, have thin basal laminae, which are permeable for gold nanoparticles, up to 15 nm in diameter. The basal laminae of tissues such as silk glands, Malpighian tubules, heart and muscles, which are not involved in the synthesis of haemolymph proteins, have thick basal laminae, which are impermeable for gold nanoparticles 6 nm in diameter. The thick basal lamina in *P.scaber* hindgut thus might be a modification related to the mechanical integrity of the hindgut that has to be adapted to a changing volume and has to withstand forces due to muscles contractions. As basal lamina is in general an integrative part of tissue permeability/barrier properties, additional data on the molecular composition would be necessary to evaluate further this issue.

## Conclusions

Epithelial cells in both hindgut regions have ultrastructural features typical of transporting epithelia. The observed ultrastructural differences indicate different transport roles in the two hindgut regions and are consistent with the proposed nutrient absorptive function of anterior chamber and transepithelial ion and water fluxes of the papillate region.

Thicker procuticle with more numerous thinner lamellae implies higher stiffness of cuticle in the anterior chamber in comparison to the papillate region. This could be linked to the intensive food processing in the anterior chamber by the contraction of the surrounding muscle layers.

The hindgut cuticle is connected to individual epithelial cells by anchoring junctions on the apical plasma membrane and fibres that protrude into the cuticular matrix, which is a similar architecture as reported for exoskeletal cuticle connections to tendon cells.

Epithelial cells in the anterior chamber have an extended apical surface in particular. This indicates extensive exchange of material between cells and the hindgut lumen, which supports the proposed nutrient absorptive function of the anterior chamber. In the papillate region, both the apical and the basal surfaces are greatly increased. This indicates extensive exchange of material with both the hindgut lumen and haemocoel, which is consistent with transepithelial transport of ions and water in the papillate region involved in osmoregulation.

The intense Na^+^/K^+^-ATPase activity at infoldings of apical plasma membrane may represent the driving force for transepithelial transport of ions and water in the papillate region.

Extensive septate junctions in *P.scaber* hindgut indicate that the paracellular transport is tightly regulated in the entire hindgut and especially in the papillate region.

The basal lamina is relatively thick in comparison to basal laminae in other tissues and probably provides mechanical support for the hindgut epithelium.

## Supplementary materials

Supplementary material 1 SI Figure 1. Boxplots depicting individual measurements of cell size and cell nuclei diameter Data type: boxplot This is an open access article distributed under the terms of the Creative Commons Attribution License (CC BY 4.0), which permits unrestricted use, distribution, and reproduction in any medium, provided the original author and source are credited.Urban Bogataj, Monika Praznik, Polona Mrak, Jasna Štrus, Magda Tušek-Žnidarič, Nada Žnidaršič

Supplementary material 2 SI Figure 2. Boxplots depicting individual measurements of cuticle thickness Data type: boxplot This is an open access article distributed under the terms of the Creative Commons Attribution License (CC BY 4.0), which permits unrestricted use, distribution, and reproduction in any medium, provided the original author and source are credited.Urban Bogataj, Monika Praznik, Polona Mrak, Jasna Štrus, Magda Tušek-Žnidarič, Nada Žnidaršič

Supplementary material 3 SI Figure 3. Boxplots depicting individual measurements of basal lamina thickness Data type: boxplot This is an open access article distributed under the terms of the Creative Commons Attribution License (CC BY 4.0), which permits unrestricted use, distribution, and reproduction in any medium, provided the original author and source are credited.Urban Bogataj, Monika Praznik, Polona Mrak, Jasna Štrus, Magda Tušek-Žnidarič, Nada Žnidaršič

Supplementary material 4 SI Figure 4. Boxplots depicting individual measurements of apical membrane labyrinth depth Data type: boxplot This is an open access article distributed under the terms of the Creative Commons Attribution License (CC BY 4.0), which permits unrestricted use, distribution, and reproduction in any medium, provided the original author and source are credited.Urban Bogataj, Monika Praznik, Polona Mrak, Jasna Štrus, Magda Tušek-Žnidarič, Nada Žnidaršič

Supplementary material 5 SI Figure 5. Boxplots depicting individual measurements of basal membrane labyrinth depth Data type: boxplot This is an open access article distributed under the terms of the Creative Commons Attribution License (CC BY 4.0), which permits unrestricted use, distribution, and reproduction in any medium, provided the original author and source are credited.Urban Bogataj, Monika Praznik, Polona Mrak, Jasna Štrus, Magda Tušek-Žnidarič, Nada Žnidaršič

Supplementary material 6 SI Figure 6. Stripcharts depicting individual measurements of the spatial density of apical membrane infoldings Data type: stripchart This is an open access article distributed under the terms of the Creative Commons Attribution License (CC BY 4.0), which permits unrestricted use, distribution, and reproduction in any medium, provided the original author and source are credited.Urban Bogataj, Monika Praznik, Polona Mrak, Jasna Štrus, Magda Tušek-Žnidarič, Nada Žnidaršič

Supplementary material 7 SI Figure 7. Stripcharts depicting individual measurements of the spatial density of basal membrane infoldings Data type: stripchart This is an open access article distributed under the terms of the Creative Commons Attribution License (CC BY 4.0), which permits unrestricted use, distribution, and reproduction in any medium, provided the original author and source are credited.Urban Bogataj, Monika Praznik, Polona Mrak, Jasna Štrus, Magda Tušek-Žnidarič, Nada Žnidaršič

## References

[B1] Berridge MJ Gupta BL (1967) Fine-structural changes in relation to ion and water transport in the rectal papillae of the blowfly, *Calliphora*. Journal of Cell Science 2: 89–112. http://jcs.biologists.org/content/2/1/89 6031010 10.1242/jcs.2.1.89

[B2] Bettica A Witkus R Vernon GM (1987) Ultrastructure of the foregut-hindgut junction in *Porcellioscaber* Latreille. Journal of Crustacean Biology 7(4): 619–623. https://www.jstor.org/stable/1548647

[B3] Bitsch C Bitsch J (2002) The endoskeletal structures in arthropods: cytology, morphology and evolution. Arthropod Structure & Development 30(3): 159–177. 10.1016/S1467-8039(01)00032-9 18088953

[B4] Blaxter ML Page AP Rudin W Maizels RM (1992) Nematode surface coats: Actively evading immunity. Parasitology Today 8(7): 243–247. 10.1016/0169-4758(92)90126-M 15463630

[B5] Boudour-Boucheker N Boulo V Charmantier-Daures M Grousset E Anger K Charmantier G Lorin-Nebel C (2014) Differential distribution of V-type H^+^-ATPase and Na^+^/K^+^-ATPase in the branchial chamber of the palaemonid shrimp *Macrobrachiumamazonicum*. Cell and Tissue Research 357(1): 195–206. 10.1007/s00441-014-1845-5 24805036

[B6] Bubel A Jones MB (1974) Fine Structure of the Gills of *JaeraNordmanni* (Rathke) [Crustacea, Isopoda]. Journal of the Marine Biological Association of the United Kingdom 54(3): 737–743. 10.1017/S0025315400022906

[B7] Buchholz C Buchholz F (1989) Ultrastructure of the integument of a pelagic Crustacean: moult cycle related studies on the Antarctic krill, *Euphausiasuperba*. Marine Biology 101(3): 355–365. 10.1007/BF00428132

[B8] Burrows M Shaw SR Sutton GP (2008) Resilin and chitinous cuticle form a composite structure for energy storage in jumping by froghopper insects. BMC Biology 6: 41. 10.1186/1741-7007-6-41 PMC2584104 18826572

[B9] Cioffi M (1984) Comparative ultrastructure of arthropod transporting epithelia. American Zoologist 24(1): 139–156. http://www.jstor.org/stable/3882759

[B10] Compere P (1991) Fine structure and elaboration of the epicuticle and the pore canal system in tergite cuticle of the land isopod *Oniscusasellus* during a moulting cycle. In: Juchault P Mocquard JP (Eds) Proceedings of the Third International Symposium on the Biology of Terrestrial Isopods.July 1990, Poitiers (France), 169–175.

[B11] Compere P (1995) Fine structure and morphogenesis of the sclerite epicuticle in the Atlantic shore crab *Carcinusmaenas*. Tissue and Cell 27(5): 525–538. 10.1016/S0040-8166(05)80061-8 18621310

[B12] Compere P Goffinet G (1987a) Ultrastructural shape and three-dimensional organization of the intracuticular canal systems in the mineralized cuticle of the green crab *Carcinusmaenas*. Tissue and Cell 19(6): 839–857. 10.1016/0040-8166(87)90024-3 18620224

[B13] Compere P Goffinet G (1987b) Elaboration and ultrastructural changes in the pore canal system of the mineralized cuticle of *Carcinusmaenas* during the moulting cycle. Tissue and Cell 19(6): 859–875. 10.1016/0040-8166(87)90025-5 18620225

[B14] Compere P Jeuniaux C Goffinet G (2004) The integument: Morphology and biochemistry (Chapter 3). In: Forest J von Vaupel Klein JC Schram FR (Eds) The Crustacea, vol.1. Brill, Leiden, 59–144.

[B15] Coruzzi L Witkus R Vernon GM (1982) Function-related structural characters and their modifications in the hindgut epithelium of two terrestrial isopods, *Armadillidiumvulgare* and *Oniscusasellus*. Experimental Cell Biology 50: 229–240. 10.1159/000163151 7117666

[B16] Dillaman R Roer R Shafer T Modla S (2013) The crustacean integument: Structure and function (Chapter 5). In: Watling L Thiel M (Eds) Functional Morphology and Diversity, vol.1. Oxford University Press, New York, 140–166.

[B17] Fabritius H Sachs C Romano Triguero P Raabe D (2009) Influence of structural principles on the mechanics of a biological fiber-based composite material with hierarchical organization: The exoskeleton of the lobster *Homarusamericanus*. Advanced Materials 21: 391–400. 10.1002/adma.200801219

[B18] Flower NE Walker GD (1979) Rectal papillae in *Muscadomestica*: the cuticle and lateral membranes. Journal of Cell Science 39: 167–186. http://jcs.biologists.org/content/39/1/167.long 528579 10.1242/jcs.39.1.167

[B19] Fukudome H (2001) A combined SEM and TEM study on the basal labyrinth of the collecting duct in the rat kidney. Archives of Histology and Cytology 64(3): 339–351. 10.1679/aohc.64.339 11575430

[B20] Ghadially FN (1997) Ultrastructural pathology of the cell and matrix, volume II. Fourth Edition. Butterworth-Heinemann, Newton, MA, 1424 pp.

[B21] Halfter W Oertle P Monnier CA Camenzind L Reyes-Lua M Hu H Candiello J Labilloy A Balasubramani M Henrich PB Plodinec M (2015) New concepts in basement membrane biology. The FEBS Journal 282(23): 4466–4479. 10.1111/febs.13495 26299746

[B22] Hames CAC Hopkin SP (1989) The structure and function of the digestive system of terrestrial isopods. Journal of Zoology 217(4): 599–627. 10.1111/j.1469-7998.1989.tb02513.x

[B23] Hassall M Jennings JB (1975) Adaptive features of gut structure and digestive physiology in the terrestrial isopod *Philosciamuscorum* (Scopoli) 1763. Biological Bulletin 149(2): 348–364. 10.2307/1540531 1203332

[B24] Hild S Marti O Ziegler A (2008) Spatial distribution of calcite and amorphous calcium carbonate in the cuticle of the terrestrial crustaceans *Porcellioscaber* and *Armadillidiumvulgare*. Journal of Structural Biology 163(1): 100–108. 10.1016/j.jsb.2008.04.010 18550385

[B25] Hild S Neues F Žnidaršič N Štrus J Epple M Marti O Ziegler A (2009) Ultrastructure and mineral distribution in the tergal cuticle of the terrestrial isopod *Titanethesalbus*. Adaptations to a karst cave biotope. Journal of Structural Biology 168(3): 426–436. 10.1016/j.jsb.2009.07.017 19632333

[B26] Holdich DM Mayes KR (1975) A fine-structural re-examination of the so-called ‘midgut’ of the isopod *Porcellio*. Crustaceana 29(2): 186–192. http://www.jstor.org/stable/20102245

[B27] Hryniewiecka-Szyfter Z Storch V (1986) The influence of starvation and different diets on the hindgut of isopoda (*Mesidoteaentomon*, *Oniscusasellus*, *Porcellioscaber*). Protoplasma 134(1): 53–59. 10.1007/BF01276375

[B28] Icely JD Nott JA (1992) Digestion and absorption: digestive system and associated organs. In: Harrison FW Humes AG (Eds) Microscopic anatomy of invertebrates.Volume 10: Decapod Crustacea. Wiley-Liss, Inc., New York, 147–201.

[B29] Izumi Y Furuse M (2014) Molecular organization and function of invertebrate occluding junctions. Seminars in Cell & Developmental Biology 36: 186–193. 10.1016/j.semcdb.2014.09.009 25239398

[B30] Jansson H-B Jeyaprakash A Coles GC Marban-Mendoza N Zuckerman BM (1986) Fluorescent and ferritin labelling of cuticle surface carbohydrates of *Caenorhabditiselegans* and *Panagrellusredivivus* Journal of Nematology 18(4): 570–574. https://www.ncbi.nlm.nih.gov/pmc/articles/PMC2618573/PMC2618573 19294228

[B31] Jonusaite S Donini A Kelly SP (2016) Occluding junctions of invertebrate epithelia. Journal of Comparative Physiology B 186(1): 17–43. 10.1007/s00360-015-0937-1 26510419

[B32] Just F Walz B (1994) Immunocytochemical localization of Na^+^/K^+^-ATPase and V-H^+^-ATPase in the salivary glands of the cockroach, *Periplanetaamericana*. Cell and Tissue Research 278(1): 161–170. 10.1007/BF00305788 7954697

[B33] Klein M Applebaum SW (1975) The surface morphology of locust hindgut cuticle. Physiological Entomology 50(1): 31–36. 10.1111/j.1365-3032.1975.tb00089.x.

[B34] Kikuchi S Matsumasa M (1993) The osmoregulatory tissue around the afferent blood vessels of the coxal gills in the estuarine amphipods, *Grandidierellajaponica* and *Melita setiflagella*. Tissue and Cell 25(4): 627–638. 10.1016/0040-8166(93)90014-C 18621251

[B35] Komnick H Stockem W (1973) The porous plates of coniform chloride cells in mayfly larvae: High-resolution analysis and demonstration of solute pathways. Journal of Cell Science 12: 665–681. http://jcs.biologists.org/content/12/3/665 4124327 10.1242/jcs.12.3.665

[B36] Kostanjšek R Avguštin G Drobne D Štrus J (2003) Morphological and molecular examination of bacteria associated with the wall of the papillate region of the gut in *Porcellioscaber* (Isopoda). In: Sfenthourakis S et al. (Eds) Crustaceana Monographs, 2. Koninklijke Brill NV, Leiden, 103–120.

[B37] Kostanjšek R Štrus J Lapanje A Avguštin G Rupnik M Drobne D (2006) Intestinal microbiota of terrestrial isopods. In: Helmut K Ajit V (Eds) Intestinal microorganisms of termites and other invertebrates.Springer-Verlag, Berlin, Heidelberg, New York, 115–132.

[B38] Kostanjšek R Štrus J Avguštin G (2007) “*Candidatus* Bacilloplasma,” a novel lineage of *Mollicutes* associated with the hindgut wall of the terrestrial isopod *Porcellioscaber* (Crustacea: Isopoda). Applied and Environmental Microbiology 73(17): 5566–5573. 10.1128/AEM.02468-06 17630315 PMC2042062

[B39] Kukulies J Komnick H (1983) Plasma membranes, cell junctions and cuticle of the rectal chloride epithelia of the larval dragonfly *Aeshnacyanea*. Journal of Cell Science 59: 159–182. http://jcs.biologists.org/content/59/1/159.long 6863407 10.1242/jcs.59.1.159

[B40] Lai-Fook J Beaton C (1998) Muscle insertions. In: Harrison FW Locke M (Eds) Microscopic anatomy of invertebrates.Volume 11B: Insecta. Wiley-Liss Inc., New York, 573–580.

[B41] LeBleu VS MacDonald B Kalluri R (2007) Structure and function of basement membranes. Experimental Biology and Medicine 232(9): 1121–1129. 10.3181/0703-MR-72 17895520

[B42] Lignot JH Charmantier-Daures M Charmantier G (1999) Immunolocalization of Na^+^,K^+^-ATPase in the organs of the branchial cavity of the European lobster *Homarusgammarus* (Crustacea, Decapoda). Cell and Tissue Research 296(2): 417–426. 10.1007/s004410051301 10382282

[B43] Locke M (1961) Pore canals and related structures in insect cuticle. The Journal of Biophysical and Biochemical Cytology 10: 589–618. 10.1083/jcb.10.4.589 13762980 PMC2225106

[B44] Luquet CM Rosa GA Ferrari CC Genovese G Pellerano GN (2000) Gill morphology of the intertidal estuarine crab *Chasmagnathusgranulatus* Dana, 1851 (Decapoda, Grapsidae) in relation to habitat and respiratory habits. Crustaceana 73(1): 53–67. http://www.jstor.org/stable/20106243

[B45] Luquet CM Genovese G Rosa GA Pellerano GN (2002) Ultrastructural changes in the gill epithelium of the crab *Chasmagnathusgranulatus* (Decapoda: Grapsidae) in diluted and concentrated seawater. Marine Biology 141(4): 753–760. 10.1007/s00227-002-0860-3

[B46] Luquet G (2012) Biomineralization: insights and prospects from crustaceans. ZooKeys 176: 103–121. 10.3897/zookeys.176.2318 PMC3335408 22536102

[B47] Mayahara H Fujimoto K Ando T Ogawa K (1980) A new one-step method for the cytochemical localization of ouabain-sensitive, potassium-dependent p-nitrophenylphosphatase activity. Histochemistry 67(2): 125–138. 10.1007/BF00493231 6249779

[B48] Mellon D (1992) Connective tissue and supporting structures. In: Harrison F Humes AG (Eds) Microscopic anatomy of invertebrates.Volume 10: Decapod Crustacea. Wiley-Liss Inc., New York, 77–116.

[B49] Miller RT (2017) Mechanical properties of basement membrane in health and disease. Matrix Biology 57–58: 366–273. 10.1016/j.matbio.2016.07.001 27435904

[B50] Moussian B (2013) The arthropod cuticle. In: Minelli A, et al. (Eds) Arthropod biology and evolution. Springer-Verlag, Berlin, Heidelberg, 171–196.

[B51] Mrak P Bogataj U Štrus J Žnidaršič N (2015) Formation of the hindgut cuticular lining during embryonic development of *Porcellioscaber* (Crustacea, Isopoda). ZooKeys 515: 93–109. 10.3897/zookeys.515.9468 PMC4525038 26261443

[B52] Neville AC (1975) Biology of the arthropod cuticle. Springer-Verlag, Berlin, Heidelberg, New York, 448 pp.

[B53] Palackal T Faso L Zung JL Vernon G Witkus R (1984) The ultrastructure of the hindgut epithelium of terrestrial isopods and its role in osmoregulation. Symposia of the Zoological Society of London 53: 185–198.

[B54] Raabe D Sachs C Romano P (2005) The crustacean exoskeleton as an example of a structurally and mechanically graded biological nanocomposite material. Acta Materialia 53: 4281–4292. 10.1016/j.actamat.2005.05.027

[B55] Raabe D Romano P Sachs C Fabritius H Al-Sawalmih A Yi S-B Servos G Hartwig HG (2006) Microstructure and crystallographic texture of the chitin-protein network in the biological composite material of the exoskeleton of the lobster *Homarusamericanus*. Materials Science and Engineering A 421: 143–153. 10.1016/j.msea.2005.09.115

[B56] Reddy JT Locke M (1990) The size limited penetration of gold particles through insect basal laminae. Journal of Insect Physiology 36(6): 397–407. 10.1016/0022-1910(90)90057-M

[B57] Shanbhag S Tripathi S (2009) Epithelial ultrastructure and cellular mechanisms of acid and base transport in the *Drosophila* midgut. Journal of Experimental Biology 212: 1731–1744. 10.1242/jeb.029306 19448082

[B58] Shires R Lane NJ Inman CBE Lockwood APM (1995) Microtubule systems associated with the septate junctions of the gill cells of four gammarid amphipods. Tissue and Cell 27(1): 3–12. 10.1016/S0040-8166(95)80003-4 18621295

[B59] Seidl B Huemer K Neues F Hild S Epple M Ziegler A (2011) Ultrastructure and mineral distribution in the tergite cuticle of the beach isopod *Tyloseuropaeus* Arcangeli, 1938. Journal of Structural Biology 174: 512–526. 10.1016/j.jsb.2011.03.005 21414408

[B60] Seidl B Ziegler A (2012) Electron microscopic and preparative methods for the analysis of isopod cuticle. ZooKeys 176: 73–85. 10.3897/zookeys.176.2294 PMC3335406 22536100

[B61] Spiegel Y McClure MA (1995) The surface coat of plant-parasitic nematodes: Chemical composition, origin, and biological role - a review. Journal of Nematology 27(2): 127–134. https://www.ncbi.nlm.nih.gov/pmc/articles/PMC2619597/19277272 PMC2619597

[B62] Storch V Štrus J (1989) Microscopic anatomy and ultrastructure of the alimentary canal in terrestrial isopods. In: Ferrara F et al (Eds) Monitore Zoologico Italiano.Monografia 4: 105–126.

[B63] Štrus J Drobne D Ličar P (1995) Comparative anatomy and functional aspects of the digestive system in amphibious and terrestrial isopods (Isopoda: Oniscidea). In: Alikhan MA (Ed.) Terrestrial isopod biology.A.A. Balkema, Rotterdam, 15–23.

[B64] Štrus J Compere P (1996) Ultrastructural analysis of the integument during the moult cycle in *Ligiaitalica* (Crustacea, Isopoda). Pflügers Archiv - European Journal of Physiology 431(6): 251–252. 10.1007/BF02346363 8739359

[B65] Štrus J Blejec A (2001) Microscopic anatomy of the integument and digestive system during the molt cycle in *Ligiaitalica* (Oniscidea). In: Kensley B Brusca RC (Eds) Isopod systematics and evolution.Crustacean Issues 13: 343–352. http://www.vliz.be/en/imis?refid=11315

[B66] Štrus J Klepal W Repina J Tušek-Žnidarič M Milatovič M Pipan Ž (2008) Ultrastructure of the digestive system and the fate of midgut during embryonic development in *Porcellioscaber* (Crustacea: Isopoda). Arthropod Structure & Development 37(4): 287–298. 10.1016/j.asd.2007.11.004 18440863

[B67] Taylor HH Taylor EW (1992) Gills and lungs: the exchange of gases and ions. In: Harrison FW Humes AG (Eds) Microscopic anatomy of invertebrates.Volume 10: Decapod Crustacea. Wiley-Liss Inc., New York, 203–293.

[B68] Towle DW Kays WT (1986) Basolateral localization of Na^+^ + K^+^-ATPase in gill epithelium of two osmoregulating crabs, *Callinectessapidus* and *Carcinusmaenas*. Journal of Experimental Zoology 239(3): 311–318. 10.1002/jez.1402390302

[B69] Vernon GM Herold L Witkus ER (1974) Fine structure of the digestive tract epithelium in the terrestrial isopod *Armadillidiumvulgare*. Journal of Morphology 144(3): 337–359. 10.1002/jmor.1051440307 30322224

[B70] Vittori M Štrus J (2014) The integument in troglobitic and epigean woodlice (Isopoda: Oniscidea): a comparative ultrastructural study. Zoomorphology 133: 391–403. 10.1007/s00435-014-0232-9

[B71] Vogt G (1994) Life-cycle and functional cytology of the hepatopancreatic cells of *Astacusastacus* (Crustacea, Decapoda). Zoomorphology 114(2): 83–101. 10.1007/BF00396642

[B72] Warburg MR Rosenberg M (1989) Ultracytochemical identification of Na^+^, K^+^ -ATPase activity in the isopodan hindgut epithelium. Journal of Crustacean Biology 9(4): 525–528. 10.1163/193724089X00548

[B73] Weihrauch D O’Donnell MJ (2015) Links between osmoregulation and nitrogen-excretion in insects and crustaceans. Integrative and Comparative Biology 55(5): 816–829. 10.1093/icb/icv013 25888942

[B74] Weis-Fogh T (1960) A rubber-like protein in insect cuticle. Journal of Experimental Biology 37: 889–907. http://jeb.biologists.org/content/37/4/889

[B75] Wright JC O’Donnell MJ Sazgarb S (1997) Haemolymph osmoregulation and the fate of sodium and chloride during dehydration in terrestrial isopods. Journal of Insect Physiology 43(9): 795–807. 10.1016/S0022-1910(97)00035-8 12770491

[B76] Wright JC Carefoot TH Albers MA (2003) Osmoregulation and salt excretion in the Ligiidae and Tylidae (Isopoda, Oniscidea). In: Sfenthourakis S, et al. (Eds) Crustaceana Monographs, 2. Koninklijke Brill NV, Leiden, 311–334.

[B77] Wright KA (1987) The nematode’s cuticle: Its surface and the epidermis: function, homology, analogy: A current consensus. The Journal of Parasitology 73(6): 1077–1083. http://www.jstor.org/stable/3282284 3325620

[B78] Ziegler A (1996) Ultrastructural evidence for transepithelial calcium transport in the anterior sternal epithelium of the terrestrial isopod *Porcellioscaber* (Crustacea) during the formation and resorption of CaCO_3_ deposits. Cell and Tissue Research 284(3): 459–466. 10.1007/s004410050606 8662354

[B79] Ziegler A (1997a) Ultrastructural changes of the anterior and posterior sternal integument of the terrestrial isopod *Porcellioscaber* Latr. (*Crustacea*) during the moult cycle. Tissue and Cell 29(1): 63–76. 10.1016/S0040-8166(97)80073-0 18627812

[B80] Ziegler A (1997b) Immunocytochemical localization of Na^+^,K^+^-ATPase in the calcium-transporting sternal epithelium of the terrestrial isopod *Porcellioscaber* L. (Crustacea). Journal of Histochemistry and Cytochemistry 45(3): 437–446. 10.1177/002215549704500311 9071325

[B81] Zimmer M (1999) The fate and effects of ingested hydrolyzable tannins in *Porcellioscaber*. Journal of Chemical Ecology 25(3): 611–628. 10.1023/A:1020962105931

[B82] Zimmer M (2002) Nutrition in terrestrial isopods (Isopoda: Oniscidea): an evolutionary-ecological approach. Biological Reviews 77(4): 455–493. 10.1017/S1464793102005912 12475050

[B83] Žnidaršič N Mrak P Tušek-Žnidarič M Štrus J (2012) Exoskeleton anchoring to tendon cells and muscles in molting isopod crustaceans. ZooKeys 176: 39–53. 10.3897/zookeys.176.2445 PMC3335404 22536098

