# Supplementary figures and images for: Comparative ultrastructure of cells and cuticle in the anterior chamber and papillate region of Porcellioscaber (Crustacea, Isopoda) hindgut
Source: Zookeys. 2018 Dec 3;(801):427–58. doi: 10.3897/zookeys.801.22395 (PMC6288245; doi:10.3897/zookeys.801.22395)

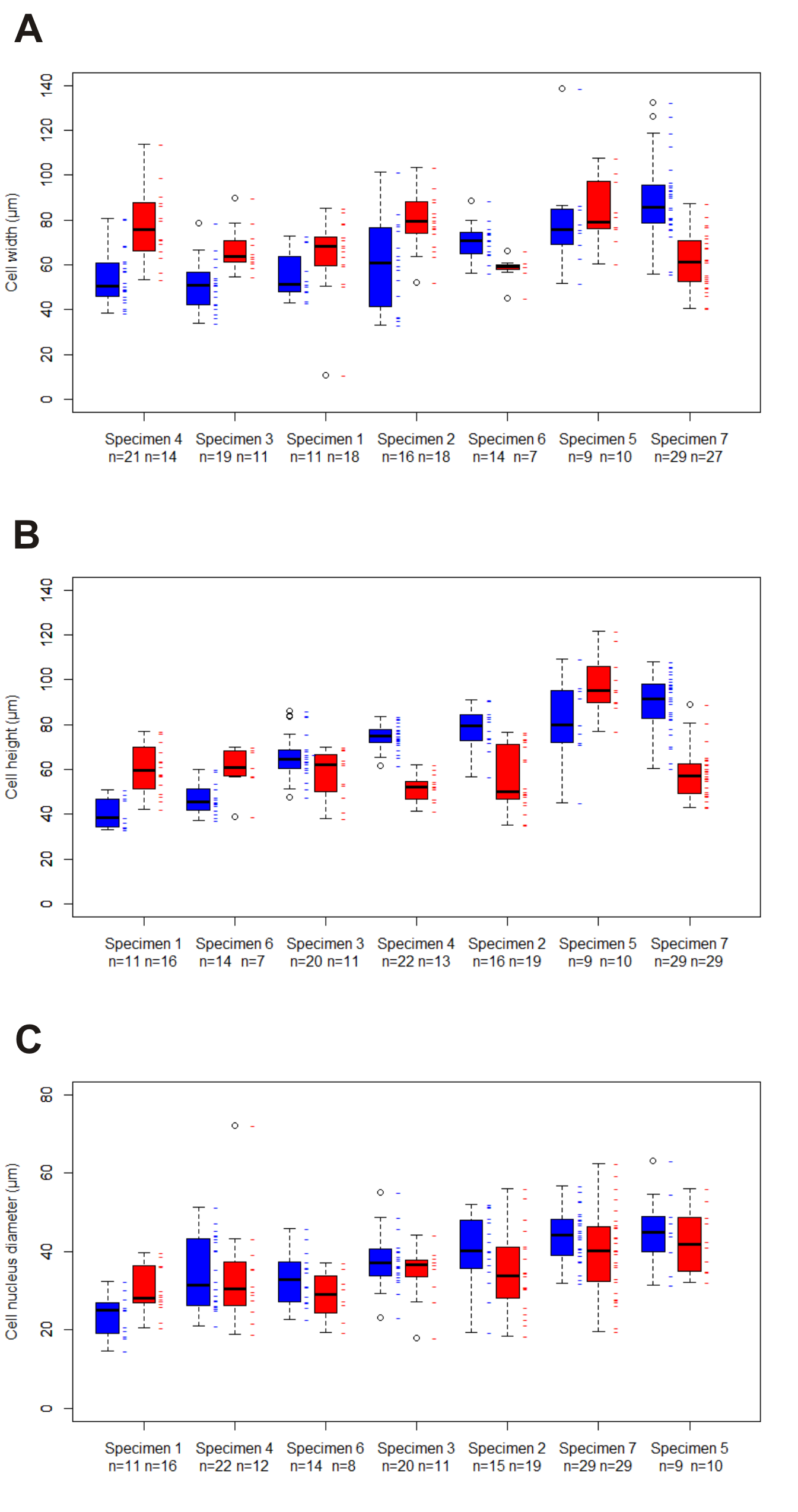

Supplement: Supplementary material 1 — SI Figure 1. Boxplots depicting individual measurements of cell size and cell nuclei diameter [file zookeys-801-427-s001.tif]

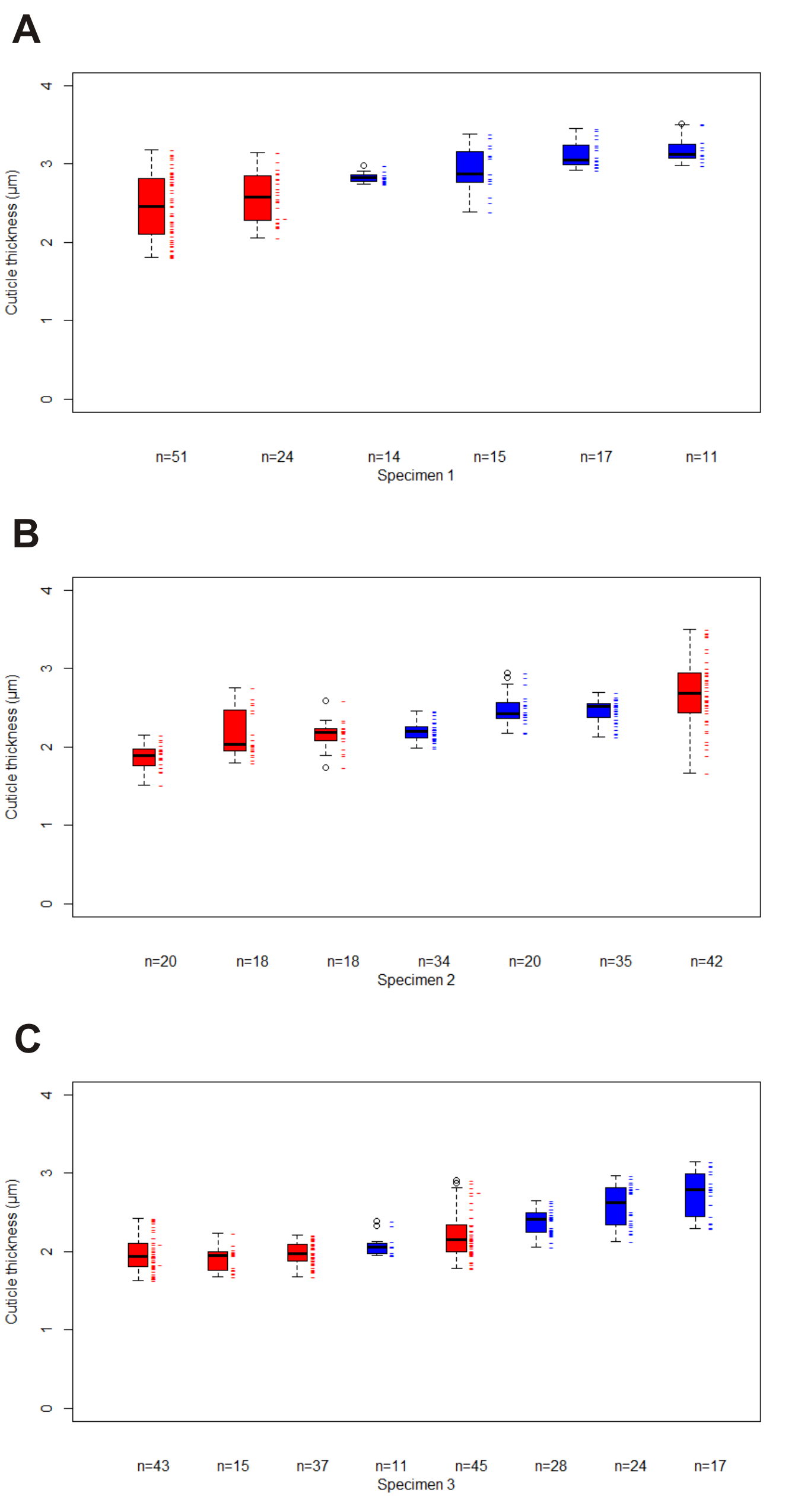

Supplement: Supplementary material 2 — SI Figure 2. Boxplots depicting individual measurements of cuticle thickness [file zookeys-801-427-s002.tif]

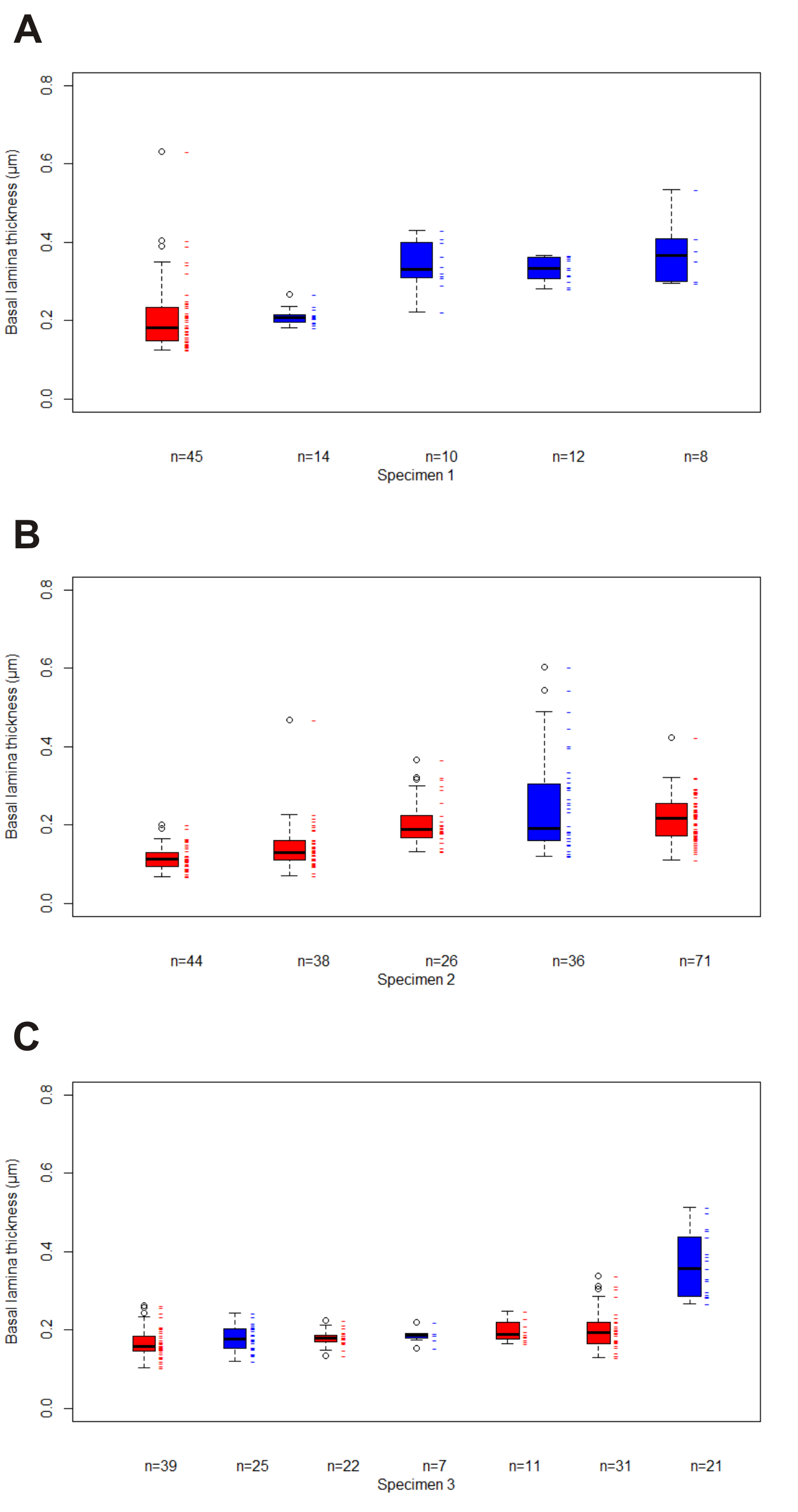

Supplement: Supplementary material 3 — SI Figure 3. Boxplots depicting individual measurements of basal lamina thickness [file zookeys-801-427-s003.tif]

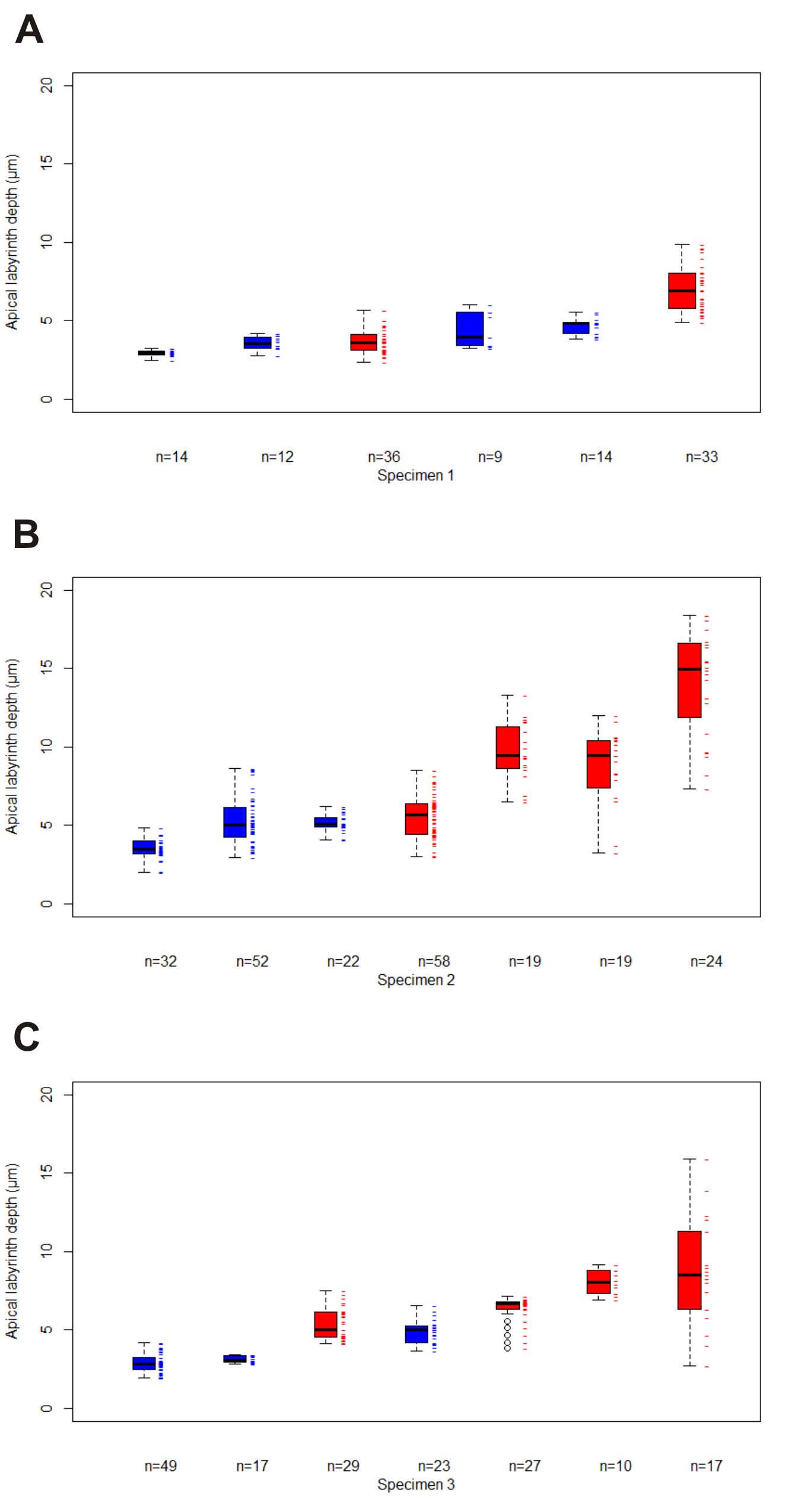

Supplement: Supplementary material 4 — SI Figure 4. Boxplots depicting individual measurements of apical membrane labyrinth depth [file zookeys-801-427-s004.tif]

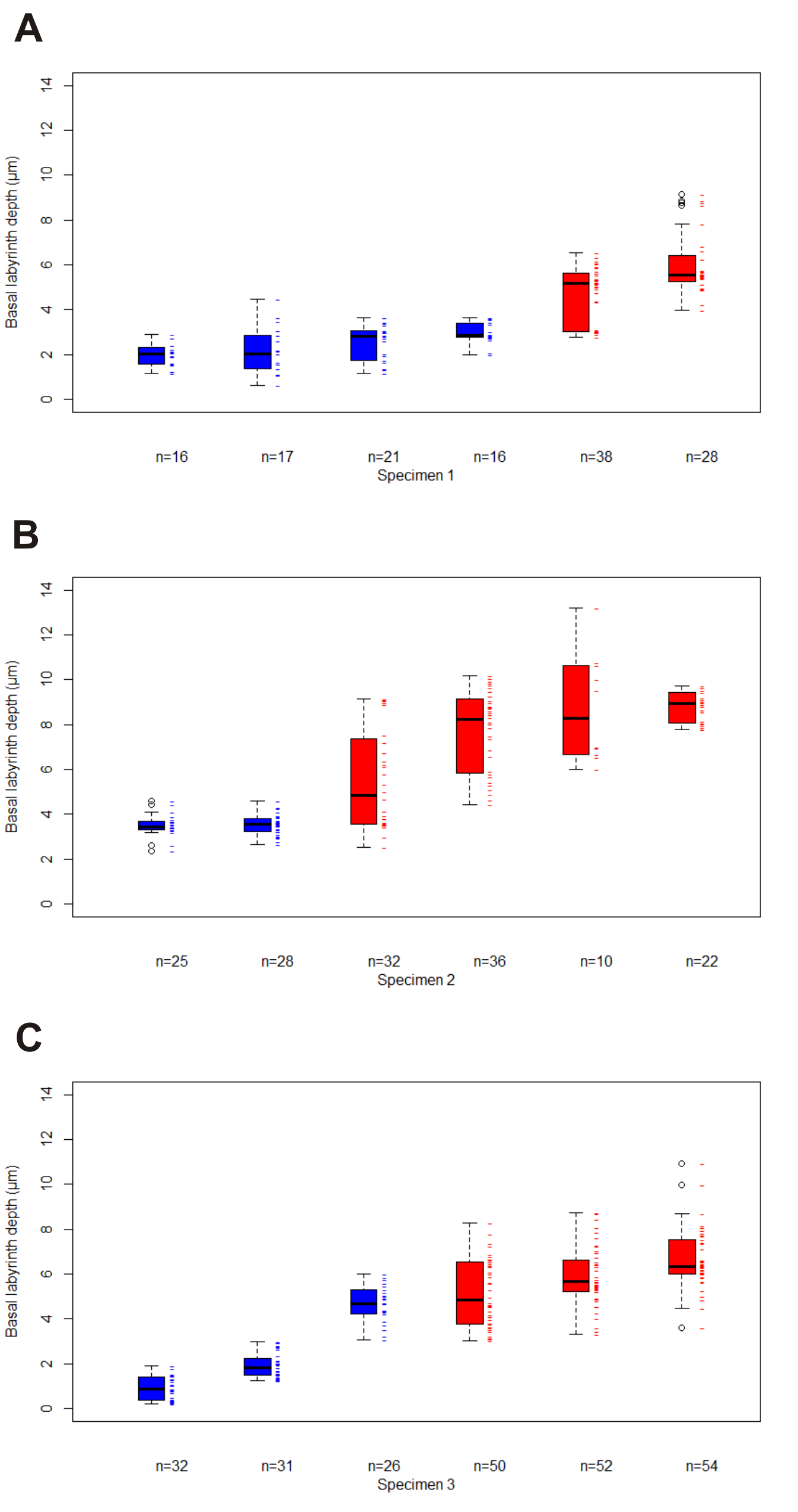

Supplement: Supplementary material 5 — SI Figure 5. Boxplots depicting individual measurements of basal membrane labyrinth depth [file zookeys-801-427-s005.tif]

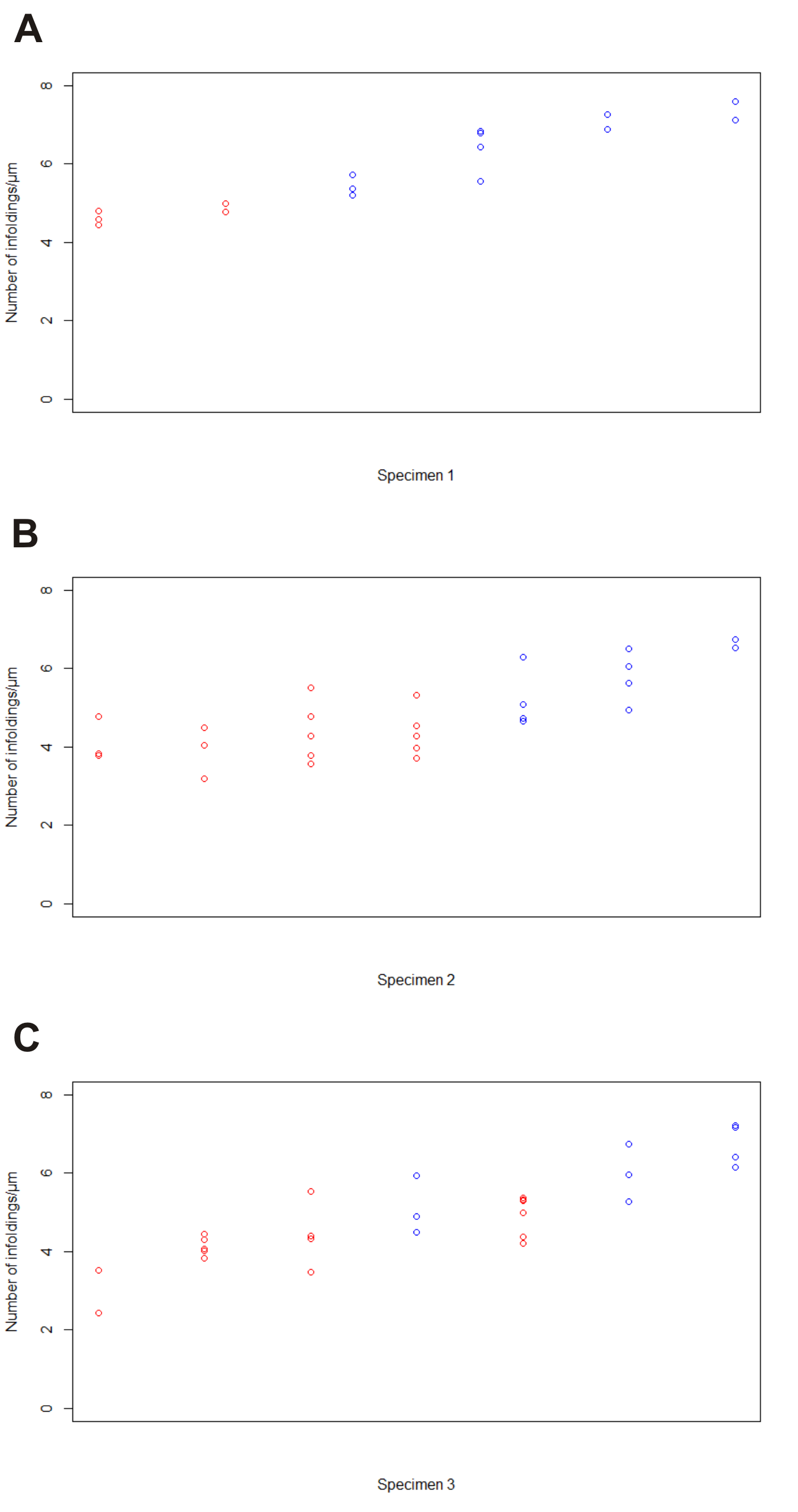

Supplement: Supplementary material 6 — SI Figure 6. Stripcharts depicting individual measurements of the spatial density of apical membrane infoldings [file zookeys-801-427-s006.tif]

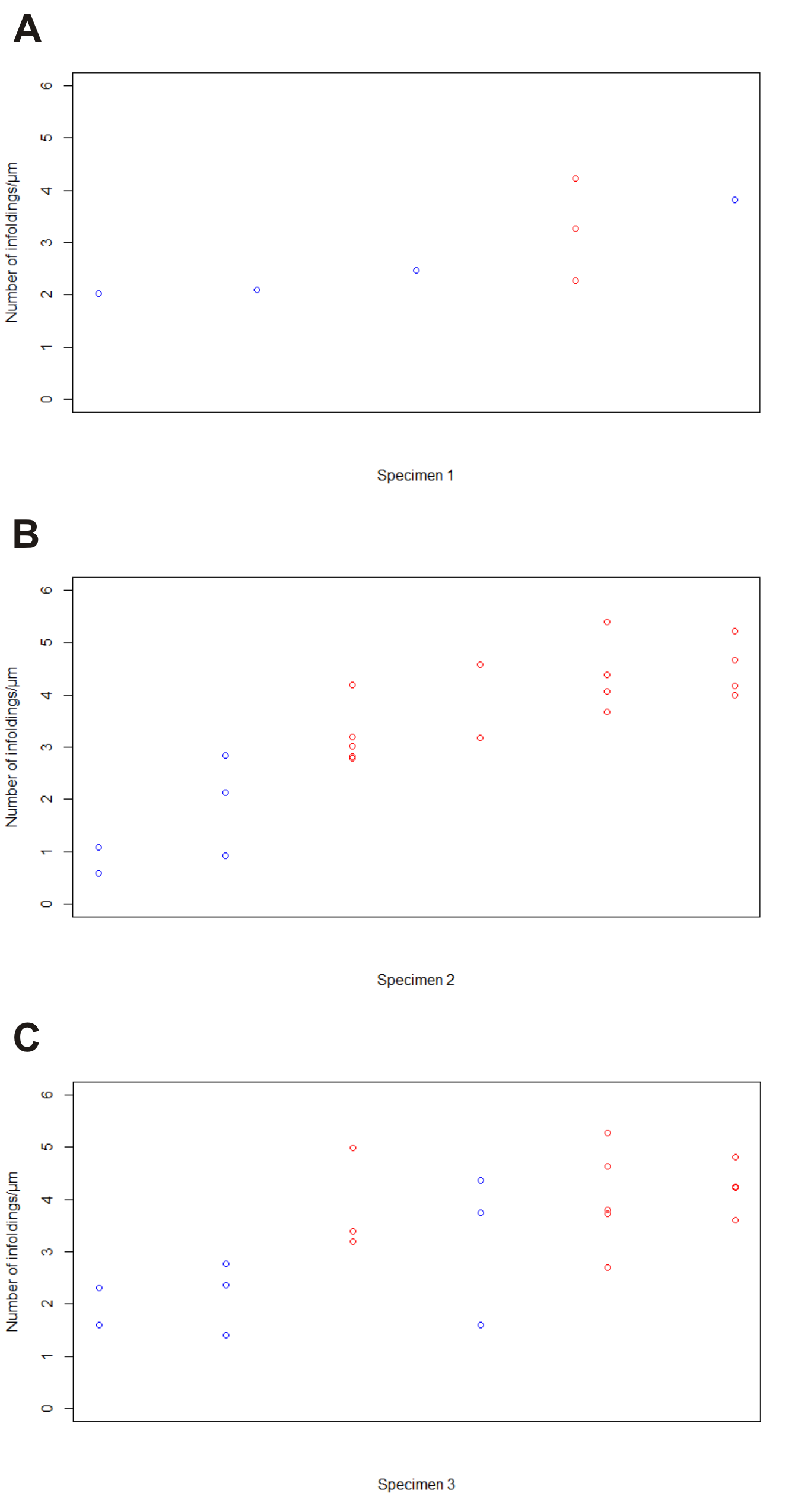

Supplement: Supplementary material 7 — SI Figure 7. Stripcharts depicting individual measurements of the spatial density of basal membrane infoldings [file zookeys-801-427-s007.tif]
